# The Assembly Switch Mechanism of FtsZ Filament Revealed by All-Atom Molecular Dynamics Simulations and Coarse-Grained Models

**DOI:** 10.3389/fmicb.2021.639883

**Published:** 2021-03-30

**Authors:** Dashuai Lv, Jingyuan Li, Sheng Ye

**Affiliations:** ^1^Life Sciences Institute, Zhejiang University, Hangzhou, China; ^2^Zhejiang Province Key Laboratory of Quantum Technology and Device, Department of Physics, Institute of Quantitative Biology, Hangzhou, China; ^3^Tianjin Key Laboratory of Function and Application of Biological Macromolecular Structures, School of Life Sciences, Tianjin University, Tianjin, China

**Keywords:** FtsZ, molecular dynamics simulation, assembly, cooperative, conformational transition

## Abstract

Bacterial cytoskeletal protein FtsZ binds and hydrolyzes GTP, and assembles into dynamic filaments that are essential for cell division. Here, we used a multi-scale computational strategy that combined all-atom molecular dynamics (MD) simulations and coarse-grained models to reveal the conformational dynamics of assembled FtsZ. We found that the top end of a filament is highly dynamic and can undergo T-to-R transitions in both GTP- and GDP-bound states. We observed several subcategories of nucleation related dimer species, which leading to a feasible nucleation pathway. In addition, we observed that FtsZ filament exhibits noticeable amounts of twisting, indicating a substantial helicity of the FtsZ filament. These results agree with the previously models and experimental data. Anisotropy network model (ANM) analysis revealed a polymerization enhanced assembly cooperativity, and indicated that the cooperative motions in FtsZ are encoded in the structure. Taken together, our study provides a molecular-level understanding of the diversity of the structural states of FtsZ and the relationships among polymerization, hydrolysis, and cooperative assembly, which should shed new light on the molecular basis of FtsZ’s cooperativity.

## Introduction

FtsZ, an ancient tubulin-like GTPase, plays pivotal roles in dividing a cell into two daughter cells (Bi and Lutkenhaus, 1991). In the presence of GTP, FtsZ subunits first polymerize into single-stranded protofilaments (Artola et al., 2017), that further coalesce into a ring-shaped pattern called Z ring (Strauss et al., 2012; Yang et al., 2017). FtsZ catalyzes the hydrolysis of GTP to GDP, which is coupled to its polymerization because the GTPase active site is completed at the interface between consecutive subunits (Mukherjee et al., 1993; Leung et al., 2004). Unlike tubulin that exhibits dynamic instability (Mitchison and Kirschner, 1984), FtsZ exhibits treadmilling (Bisson-Filho et al., 2017; Yang et al., 2017), which further directs the septal cell wall synthesis and cell division (Bisson-Filho et al., 2017; Yang et al., 2017). Treadmilling, characterized by polymerization at one end (i.e., plus end) and depolymerization at the other end (i.e., minus end), is coupled to GTPase activity and the GTPase-regulated conformational change of FtsZ subunits (Chen and Erickson, 2011; Li et al., 2013; Wagstaff et al., 2017).

FtsZ subunit adopts in two distinct conformations: an open-cleft conformation (high affinity, T state) and a closed-cleft conformation (low affinity, R state) (Fujita et al., 2017). Although two different conformations have been observed in both free subunit and filaments from different organisms, and with GTP or GDP bound (Oliva et al., 2007; Matsui et al., 2014; Fujita et al., 2017; Wagstaff et al., 2017), considerable controversy remains regarding the effect of nucleotide on the conformational change of FtsZ subunit. These results have led to two different mechanisms for the conformational changes. The “hydrolyze and bend” model proposed that the protofilament is straight with GTP- and curved with GDP-bound (Allard and Cytrynbaum, 2009). This model has been supported by both biochemical and theoretical studies. In the presence of GTP, purified FtsZ adopts either straight or intermediate curved filaments. With the hydrolysis of GTP to GDP, highly curved filaments form (Erickson et al., 1996; Lu et al., 2000; Huecas and Andreu, 2004). Molecular dynamics (MD) simulations have shown different curvatures of FtsZ filaments that are supported by a nucleotide-regulated hinge motion between subunits (Hsin et al., 2012; Ramírez-Aportela et al., 2014). Crystallographic study of the *Mycobacterium tuberculosis* FtsZ (MtbFtsZ) proposed that hydrolysis of GTP triggers a hinge-opening motion of the FtsZ subunits, thereby leading to a straight-to-curved conformational change of FtsZ filaments (Li et al., 2013). By contrast, a structural comparison of *Staphylococcus aureus* FtsZ (SaFtsZ, PDB entries: 3VOA) (Matsui et al., 2012) that adopt an open-cleft conformation in the straight filament with *Bacillus subtilis* FtsZ (BsFtsZ, PDB entry: 2RHL) (Raymond et al., 2009) that adopt an closed-cleft conformation in free subunit implies that the change of subunit conformation is affected by the structure change of FtsZ filament rather than the bound nucleotide (i.e., a polymerization-associated model). Thus, the exact role of the nucleotide in the conformational changes of FtsZ remains controversial.

In addition, the cooperative assembly of FtsZ is a prerequisite for the treadmilling of the single-stranded protofilaments. In a cooperative polymerization, the assembly of a single-stranded protofilament was proposed to have two stages: an unfavorable weak dimer or trimer nucleation followed by a more favorable elongation (Huecas et al., 2008; Miraldi et al., 2008). However, it raises a previously posed question of how a single-stranded protofilament with only one type of bond can assemble in an apparently cooperative manner. A “polymerization-associated” model was recently proposed by Wagstaff et al. to enable such a manner if the assembly switching of the R subunits (corresponding to a free subunit) into the T subunits (corresponding to the polymerized subunit) is coupled to the formation of a tight association interface between consecutive subunits along the filament (Wagstaff et al., 2017). Similarly, Miraldi et al. have proposed some possible nucleation pathways and all of which occurred of two R subunits to form a T-T dimer (Miraldi et al., 2008). Dajkovic et al. proposed a related model in which two R subunits switched to T and then associated to make a T-T dimer (Dajkovic et al., 2008). Recently Corbin and Erickson have presented a much more detailed model and suggested that nucleation occurrs when a T subunit binds an R subunit which then switches to T, resulting in a T-T dimer (Corbin and Erickson, 2020). All of the proposed models seem to work and shed light on several aspects of FtsZ assembly dynamics, but more data, especially those at the molecular-level, are needed to identify the feasible nucleation pathway from the previously proposed ones.

Along with many other studies, Ramirez-Aportela et al. suggested that a dimer or a trimer may be enough to produce the first polymerization nucleus (Chen et al., 2005; Huecas et al., 2008; Lan et al., 2008; Miraldi et al., 2008; Martín-Galiano et al., 2010; Ramírez-Aportela et al., 2014). Moreover, Corbin and Erickson proposed that if the penultimate subunit has a GDP, this interface is considerably weakened to permit the terminal subunit to switch to low affinity conformation and dissociate. As suggested by these studies (Corbin and Erickson, 2020), the middle subunit in a trimer is maintained in the T conformation regardless of GTP or GDP. Only a terminal subunit with GDP can switch to R. In order to better understand the cooperative mechanism of the protofilament assembly, the computational study about the conformation dynamics of FtsZ guided by nucleotide hydrolysis and polymerization is highly warranted.

Molecular dynamics (MD) simulation is suitable to investigate such a process. Due to the limit of computation compacity, it is difficult for MD simulations to address the process with the milliseconds-to-seconds timescales like the formation of functional FtsZ protofilaments (less than 20 subunits *in vivo* and 30–50 *in vitro*) (Romberg et al., 2001; Walker et al., 2020). Simplified coarse-grained models such as gaussian network model (GNM) and the anisotropic network model (ANM) (Bahar et al., 1997a,b; Atilgan et al., 2001; Wang et al., 2019) have been established as valid and efficient means to probe the large-scale collective motions relevant to protein functions and flexibility.

In this work, we investigated the assembly dynamics of a MtbFtsZ trimer in different nucleotide-binding states using both all-atom MD simulations and coarse-grained models. Our study provides a molecular-level understanding of the diversity of the structural states of FtsZ and the relationships among polymerization, hydrolysis, and cooperative assembly. The results allow us to fit the previously published models, and to identify the feasible nucleation pathway.

## Materials and Methods

### Homology Modeling of the FtsZ Trimer

Li et al. (2013) found that a biologically relevant MtbFtsZ protofilament exhibits a curved conformation, adopting the opposite curvature toward the membrane. Such a conformation is at odds with the known membrane-facing geometry of the C terminus of the FtsZ filaments (Osawa et al., 2009). To probe whether MtbFtsZ can also adopt the curvature toward the membrane, we performed MD simulations based on MtbFtsZ. As the high affinity conformation of MtbFtsZ has not been crystallized, we constructed the three-dimensional structure of this state using Swiss-Model (Waterhouse et al., 2018)^1^ with the corresponding wild-type structure of SaFtsZ (Fujita et al., 2017) bound to GDP as a template (2.2 Å resolution, chain A, PDB code: 5H5G), which is an appropriate choice because of the high sequence identity (63.28%) between the target sequence and the template as well as the high structural similarity (i.e., the RMSD of C_α_ atoms between the low affinity conformations of MtbFtsZ (PDB entry 5ZUE, residues 9-312) (Guan et al., 2018), and SaFtsZ (PDB entry 3WGK, residues 12-315) (Matsui et al., 2014) is less than 0.9 Å). The GTP coordinate was obtained from MtbFtsZ-GTP (PDB entry 5zue) (Guan et al., 2018). The GTP- and GDP-bound trimers were constructed based on crystallographic symmetry from the SaFtsZ structure (PDB code: 5H5G), and the subunit segment residues from Ala^9^ to Phe^312^ were used for simulations. The quality of the homology model was assessed by Ramachandran plot generated from PROCHECK program with 99.6% residues in the allowed regions (Laskowski et al., 1993). The stereochemistries of the models were further improved using a typical equilibrated protocol (Phillips et al., 2005) (see details below).

### Systems Preparation

Two simulations systems were constructed: GTP- and GDP-bound trimers. Amber force fields ff14SB and Amber parameter database from tLeap of AmberTools 18 were used to describe the interaction of the protein and ligands (GDP and GTP) (Meagher et al., 2003; Case et al., 2005). Each trimer was immersed in cuboid boxes of TIP3P (Jorgensen et al., 1983) water molecules. The boxes were replicated by the periodic boundary conditions. All systems were neutralized by randomly placing a proper number of sodium cations in the simulation boxes.

### Molecular Dynamics Simulations

All simulations were performed using the NAMD 2.12 package (Phillips et al., 2005). All systems were simulated in the NPT ensemble conditions. The temperature was maintained at 298 K by the Langevin thermostat, and the pressure was kept at 1.013 bar using the Nosé-Hoover Langevin piston control (Feller et al., 1995). The covalent bonds involving hydrogen atoms were constrained using the SHAKE algorithm (Miyamoto and Kollman, 1992), which allows the usage of a time step of 2 fs. The short-range non-bonded interactions were truncated at 12 Å. Meanwhile, the long-range electrostatic interactions were treated via the particle-mesh Ewald method (Darden et al., 1993) with a grid spacing of 1 Å. The system was equilibrated using the following sequence of steps: First, energy minimization was performed using steepest descent and conjugated gradient algorithm for 20000 steps. Then, the system was slowly heated from 50 K to the target 298 K over 6 ns through increasing the temperature by 50 K every 1 ns with the ligands and protein restrained. Thirdly, equilibration was continued for another 6 ns with only C_α_ atoms of protein restrained. Two 150 ns independent production runs were performed for the GTP-bound trimer. As for the GDP-bound trimer, three 450 ns independent production MD simulations were performed to obtain reliable results of the conformations of more flexible GDP-bound trimer.

Although larger-sized filaments and longer timescales (several microseconds or more) should better reproduce the macroscopic mechanics of FtsZ filaments. Two reasons lead us to believe that our simulations sampled different representative conformations of the FtsZ filaments. First, two GTP-bound simulations and two of three GDP-bound simulations showed consistent dynamical behavior of the subunit interfaces. The subunits’ conformations in the GTP-bound trimer tend to maintain in T state, while those in the GDP-bound trimer tend to switch to R state. Second, our simulation times are similar with previous dimeric and heptameric filament studies (Hsin et al., 2012; Ramírez-Aportela et al., 2014), where they suggested the simulations on FtsZ were quite stable after 200 ns and the GTP state can be much more stable than GDP state. We have test this by extending one GTP-bound simulation to 300 ns, and no differences between the 150 ns and 300 ns simulations were observed in our GTP-bound state (Supplementary Figure 1). Two things should be noted. First, in some figures we scaled timescale of these 150 ns and 450 ns simulation to a range of 0 to 1 for easier comparison, and used “reaction coordinate” to represent it, e.g., 0 represent 0 ns and 1 represented 150 ns and 450 ns for GTP and GDP state, respectively. Second, we analyzed the results by averaging all the independent MD simulations to grasp the average behavior of FtsZ filament dynamics. Meanwhile, we further analyzed all independent trajectories to get the detailed order of switching of each subunit in the trimer and fit the results into the previously published models.

### SVD Analysis of the MD Trajectories

Singular value decomposition (SVD) analysis of the MD trajectories provides an effective means for separating different conformations (Ren, 2016). SVD of C-alpha atom’s distance matrices of FtsZ obtained from MD trajectories was performed in each system using Matlab. Because the first few singular vectors (here refer as principal components, PCs) provide useful description of subpopulations. Thus, we performed FEL analysis (Tripathi et al., 2018) to compare the independent trajectories in one conformation space by calculating joint probability distribution from essential plane made by top two PCs obtained from the SVD analysis of the MD trajectories.

### Linear Regression

Linear regressions were calculated by the LinearModel class in Matlab. F-statistic were used for the observed *p*-values of linear regressions, where the null hypothesis is a zero coefficient of regression. The *p*-values were adjusted to represent a sample size corresponding to a 1-ns interval between independent states for the simulation (Ng et al., 2019).

### Calculation of Buried Solvent-Accessible Surface Area

The buried SASA between subunits can be expressed as:

(6) buriedSASA=(M+1M-2M)1+2/2

where M_1_ and M_2_ represent the SASA of each subunit, M_1+2_ represent the SASA of the complex of the two subunits. More details regarding the buried SASA calculations have been described in previous work (Hsin et al., 2012).

### Cross Correlations Between Atomic Fluctuations

To remove the border effects that are induced in MD simulations while without the expense of removing the end subunits, we calculated the correlation between residue fluctuations based on ANM method (ANM source codes obtained from Bahar lab)^2^, which is exclusively based on inter-residue contacts without the consideration of environment. In the studies, the final simulated GTP- and GDP-bound structures were chosen for ANM studies. A cutoff distance of 15 Å is adopted to include a somewhat broader range of interactions. And the correlations are obtained over 45 modes of motion. The values of pair-wise cross-correlations between residues (C*_*ij*_*) are between –1 and 1. The positive values represent the motions in the same direction, and the negative values illustrate the residues move in the opposite direction (Figure 11H). The higher the absolute value is, the more the two residues are correlated. The value *C*_*ij*_ = 0 means that the motions of residues are completely uncorrelated.

## Results

### Comparison of the Independent Simulations

**FIGURE 1:**
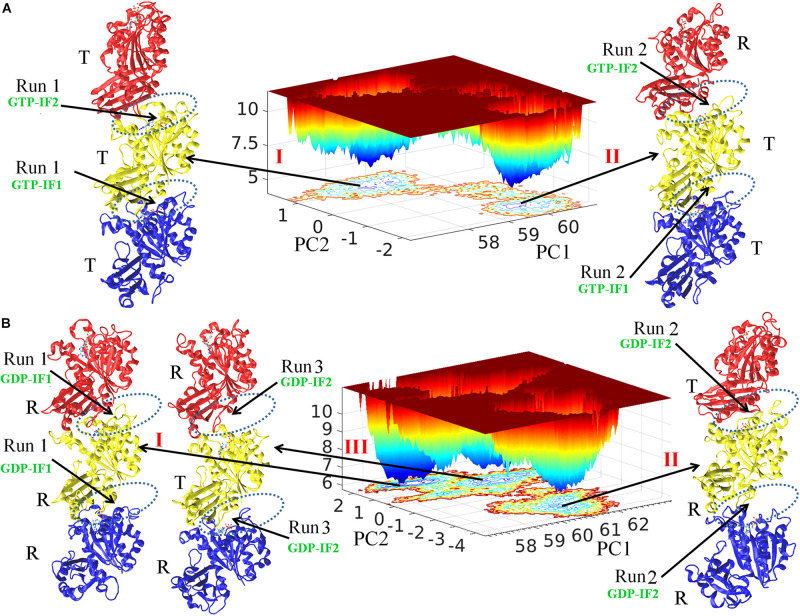
Free energy landscape and the structural analysis. FEL plot of GTP- **(A)** and GDP-bound **(B)** trimers during whole production simulations. The final conformations from independent simulations were named I, II and III. The interfaces are indicated by dashed ellipses. The bottom, middle and top subunit are colored with blue, yellow and red, respectively. PC1 and PC2 are the first and second singular vectors obtained from SVD analysis, respectively. The *z* axis corresponds to free energy (*k*_*B*_T).

Free energy landscape analysis (see Methods) was performed to study both GTP- and GDP-bound states (Figure 1). There are two and three energy funnels for GTP- and GDP-bound states, respectively. The energy barriers between the energy funnels in the GTP-bound state are higher, whereas the GDP-bound state has broader local energy minima and can access multiple states for both trimer and subunit (Figure 1 and Supplementary Figure 2). We found that the conformations with lowest energy in the five funnels correspond to the structures obtained from the final five MD trajectories (two and three for GTP- and GDP-bound states, respectively). The final simulated structures were shown around the corresponding funnels. We monitored the association strength between subunits by calculating inter-subunit’s buried SASA (Supplementary Figure 3), and observed a clear gap between the buried SASAs of the open and closed interfaces. We hereafter denote the closed interface with a value of buried SASA higher than 1100 Å^2^, and the open interface with a value lower than 1000 Å^2^, respectively. In addition, we monitored the subunit’s T to R transition by calculating inter-domain’s distances during every MD trajectory (Supplementary Figure 4) and also observed a clear gap between the inter-domain distances of the T and R states. We therefore denote the R state with a reduction of the inter-domain distance more than 2.5 Å than that of the T state. We hereafter use R and T to refer the subunit conformational states, and open and closed to refer the interfaces. Supplementary Videos 1–4 show the time evolutions of the interfaces and the conformations in the GTP- and GDP-bound states by snapshots (time-averaged structures), respectively. Supplementary Figure 5 shows the comparison of every subunit within structures in Figure 1 with the initial subunit in T state and the crystallographic structure of MtbFtsZ (PDB ID 4KWE) subunit in R state.

We observed both homogeneous and heterogeneous distributions of conformations for both subunits (T and R conformations) and interfaces (open and closed conformations) in the FtsZ trimers. The structure I and II obtained from the final GTP-bound trajectories share structural features with the top (binding nucleotide) interface open (GTP-interface 2, GTP-IF2) and the bottom one closed (GTP-interface 1, GTP-IF1) (Figure 1A and Supplementary Figure 3). The subunits in GTP-bound structure I form a homogeneous T-T-T (bottom-middle-top) trimer and the subunits in GTP-bound structure II form a heterogeneous T-T-R (bottom-middle-top) trimer (Figure 1A and Supplementary Figure 4). On the other hand, structure II and III of the final GDP-bound trajectories share a distinct structural feature with both interfaces open (GDP-interface 2, GDP-IF2), albeit structure I is in a somewhat closed interface (GDP-interface 1, GDP-IF1) (Figure 1B and Supplementary Figure 3). The subunits in GDP-bound structure I, II and III form R-R-R (bottom-middle-top, homogeneous), R-R-T (bottom-middle-top, heterogeneous) and R-T-R (bottom-middle-top, heterogeneous) trimers, respectively (Figure B and Supplementary Figure 4). Overall, the GDP-bound filaments revealed a higher conformational variability, whereas the GTP-bound filaments adopted rigid conformations. Such a nucleotide-regulated conformational variability agrees with previous studies (Hsin et al., 2012; Ramírez-Aportela et al., 2014). Although both GTP- and GDP-bound states can exhibit a heterogeneous distribution of T and R conformations in trimers, we observed that the T conformations tend to be adopted by GTP-bound FtsZ, while the R conformations by GDP-bound FtsZ. Next, to obtain useful information from the simulations, both the average behavior from all parallel trajectories and also the specific behavior in individual trajectory were analyzed in each case. Importantly, we further discuss the heterogeneous distribution of T and R conformations in trimers and fit the results into the previously published models.

### Bending Flexibility and Dynamics of a FtsZ Trimer

**FIGURE 2:**
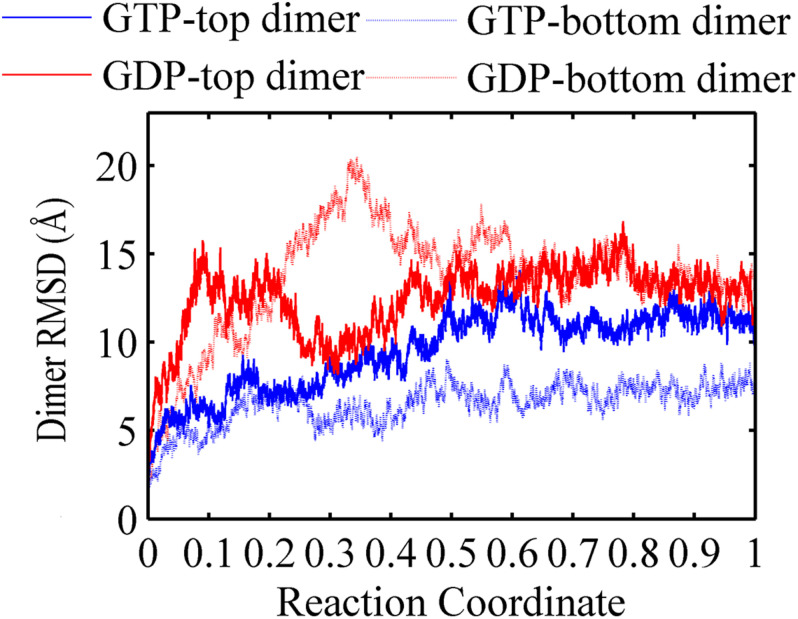
Root mean square deviations of the C_α_ atoms of dimers from the initial conformation as a function of time. Blue solid line: GTP-bound top dimer. Blue dashed line: GTP-bound bottom dimer. Red solid line: GDP-bound top dimer. Red dashed line: GDP-bound bottom dimer. Reaction coordinate: 0 represent 0 ns and 1 represented 150 ns and 450 ns for GTP and GDP state, respectively. The RMSDs are from a starting zero for initial dimer conformation. In all the cases, the RMSDs are calculated by averaging all of the GTP-bound (two runs) or GDP-bound (three runs) trajectories.

A FtsZ filament asymmetrically contains two ends. To understand what happens to the top interface in the trimer when there is no subunit above the top, and to the bottom interface with no subunit below, we used the root mean square deviations (RMSDs) of the C_α_ atoms of dimers to measure the structural changes of the top and bottom dimers during the simulation (Figure 2). As shown in Figure 2, both RMSDs of the top (binding nucleotide) and bottom dimers of a GDP-bound trimer were ∼11 Å (red solid and dashed lines). By contrast, the RMSDs of the dimers in GTP-bound trimer are asymmetric: the RMSD of the bottom dimer was ∼7 Å (Figure 2, blue dashed line), while that of the top one was ∼13 Å (Figure 2, blue solid line).

**FIGURE 3:**
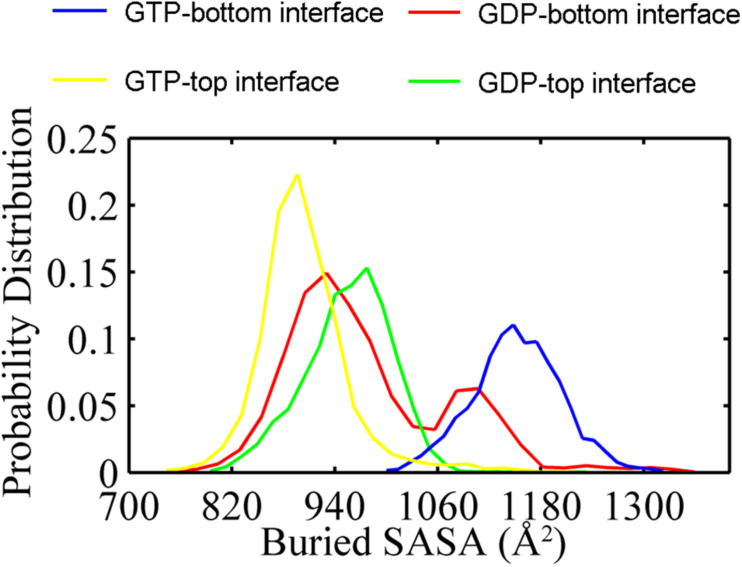
Distribution of buried SASA values for the GTP- (yellow) and the GDP-bound (green) top interfaces, and for the GTP- (blue) and the GDP-bound (red) bottom interfaces. The interfaces start from the same value about 1200 Å^2^. In all the cases, the buried SASAs are calculated by averaging all the GTP-bound (two runs) or GDP-bound (three runs) trajectories.

In addition, the buried SASAs of the dimers revealed a similar trend while with more details. The GTP-bound bottom dimer tends to maintain a high buried SASA of ∼1100 Å^2^ (Figure 3, blue line and Supplementary Figure 6A), indicating a stronger inter-subunit interaction and a more compact interface (closed interface). By contrast, the buried SASA for the GTP-bound top interface reduces to ∼900 Å^2^ (Figure 3, yellow line and Supplementary Figure 6A), indicating the tendency to form a weakened inter-subunit interaction (open interface). As for the case of GDP-bound trimer, the top and bottom interfaces reached similar buried SASA values of ∼980 Å^2^ and ∼930 Å^2^ (Figure 3, red and green lines and Supplementary Figure 6B), indicating the tendency to form weak inter-subunit interactions. Nevertheless, the buried SASA values of the GDP-bound top interface significantly reduces in the early stage of simulation (Supplementary Figure 6B, blue line), while the bottom interface experiences a period of relatively large buried SASA for nearly 100 ns, fluctuating around 1100 Å^2^. At ≈ 100 ns, the buried SASA of the GDP-bound bottom interface continuously reduces (Supplementary Figure 6B, red line). These results demonstrate that the intrinsic structure dynamic of the top interface without subunit above in the filament is considerable, and thus more prone to disassembly. The dynamic difference between the top and bottom interface leads us to suggest that the top end of FtsZ filament to be the kinetic minus end and the bottom end to be the plus end. This agrees well with the mutagenesis studies (Du et al., 2018).

**FIGURE 4:**
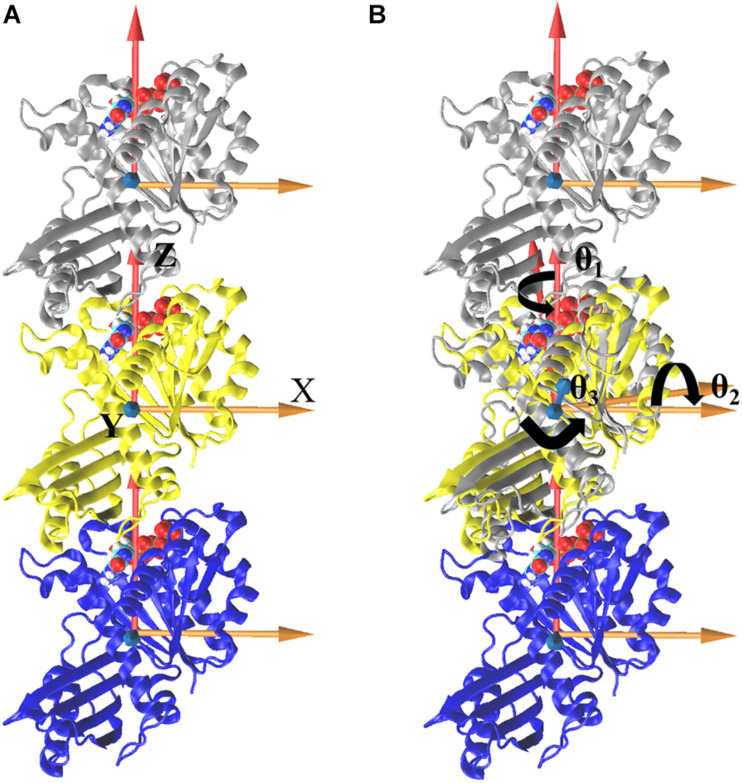
Definitions of bending and twisting angles. Overall, the coordinate system was defined by aligning the *z* axis along the center of mass position of the one subunit to that of another subunit, fixing the *x* axis along a preferred bending direction for the trimer and defining an orthogonal *y* axis **(A)**. To measure the relative motions, trimer was first aligned with respect to the initial straight frame by superposing the middle subunits (yellow). The bending angles, θ_1_ (rotations around the *Z* axis), θ_2_ (rotations around the *X* axis), and θ_3_ (rotations around the *Y* axis), were then tracked by calculating the rotations of the top (silver) subunit to align the initial reference frame of the middle subunit (**B**, align top subunit as an example). The same procedure is performed for bottom subunit, and the right-handed coordinate system is used here.

**FIGURE 5:**
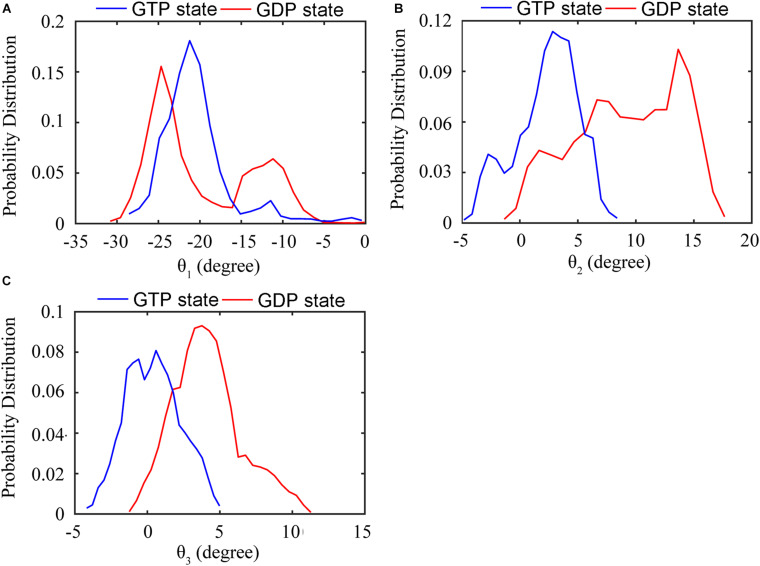
Distribution of the relative rotation angles between subunits in the GTP- (blue) and GDP-bound (red) trimers. **(A)** Twisting motion around θ_1_. **(B)** Bending motion around θ_2_. **(C)** Bending motion around θ_3_. In all the cases, the rotation angles are calculated by averaging both top and bottom interfaces over all GTP-bound (two runs) or GDP-bound (three runs) trajectories.

We further analyzed the averaged rotation angles of the subunit-subunit interfaces (see Figure 4 for details). Both GTP- and GDP-bound trimers exhibit noticeable bending flexibility (Supplementary Figures 7A–C). The probability distributions (Figures 5A–C) revealed a higher bending flexibility in the GDP-bound state (θ_1_ = −24.7°, θ_2_ = 13.7° and θ_3_ = 3.8°) than that in the GTP-bound state (θ_1_ = −21.2°, θ_2_ = 2.8° and θ_3_ = 0.6°), and the preferred bending modes are along θ_1_ and θ_2_. Rotation around θ_2_ matches previously proposed direction to generate bending forces on the membrane (Osawa et al., 2009) with the C terminus on the outside. But our simulated bending direction (θ_2_) is opposite to the direction in the crystal structure (Li et al., 2013), indicating that MtbFtsZ can bend in two opposite directions. The above twisting angle (θ_1_) reflects the mean twisting flexibility in each case. In fact, we observed both homogeneous and heterogeneous interfaces in FtsZ trimers. In Supplementary Figure 8, we calculated the distributions of twist angles (θ_1_) in four different types of interfaces. The highest probability values of θ_1_ were −6.1°, −9.4°, −33.1° and −35.7° for GTP-IF1, GDP-IF1, GDP-IF2 and GTP-IF2, respectively. The ability of the FtsZ filament to access at such a large range of θ_1_ in our simulations suggests a substantial plasticity of the FtsZ filament.

### Coupled Motions Between Inter-domain and Inter-subunit

**FIGURE 6:**
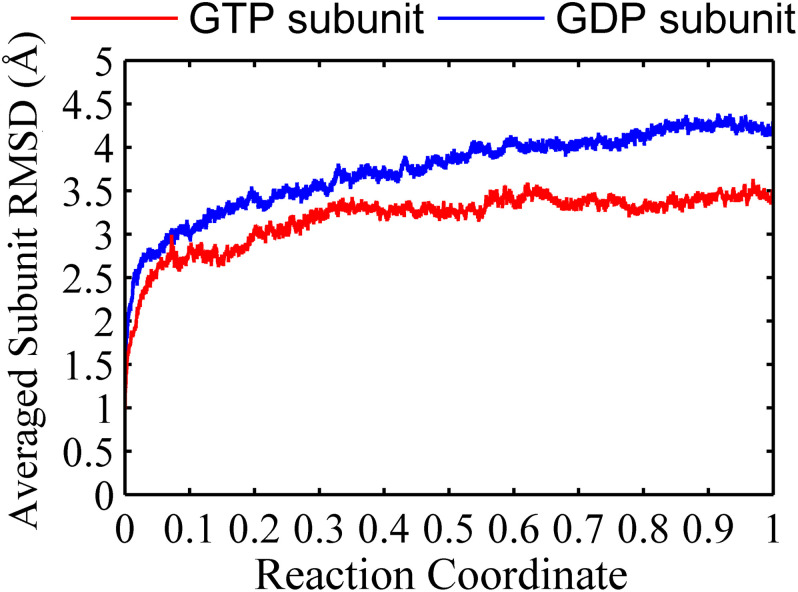
Root mean square deviations of the C_α_ atoms of subunits from the initial conformation as a function of time. Red line: GTP-bound subunit. Blue line: GDP-bound subunit. Reaction coordinate: 0 represent 0 ns and 1 represented 150 ns and 450 ns for GTP and GDP state, respectively. The RMSDs are from a starting zero for initial subunit conformation. In all the cases, the RMSDs are calculated by averaging all of the three subunits of trimers in GTP-bound (two runs) or GDP-bound (three runs) trajectories.

**FIGURE 7:**
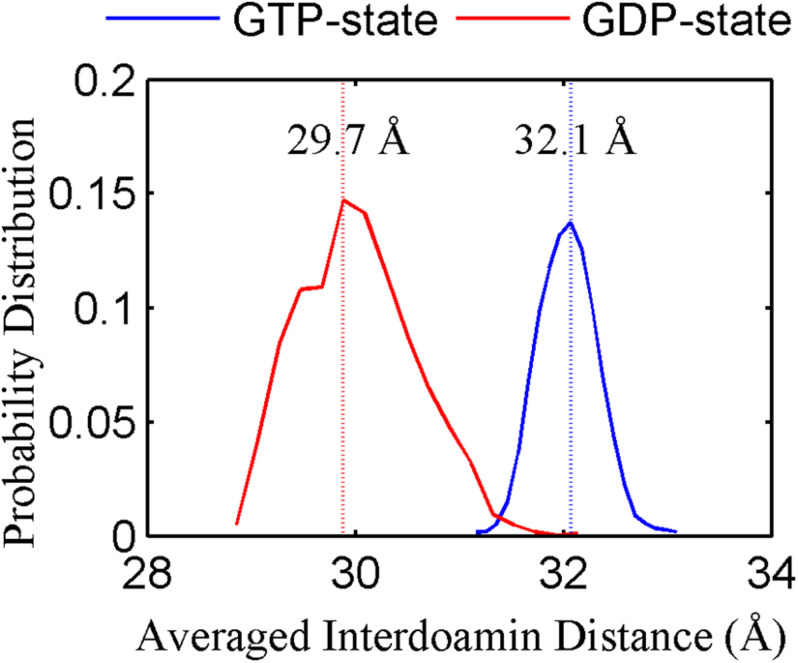
The inter-domain distance distributions for the GTP- (blue) and GDP-bound (red) states. In all the cases, the distances are calculated by averaging all the GTP-bound (two runs) or GDP-bound (three runs) trajectories.

**FIGURE 8:**
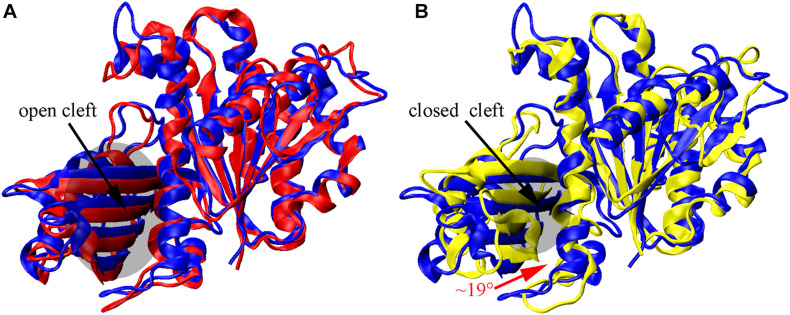
Nucleotide-regulated subunit conformational changes. **(A,B)** superimposition of the N-terminal domain of the initial T structure (blue) on monomeric structures obtained by averaging over all three subunits from the final 10 ns GTP- (red) and GDP-bound (yellow) simulations. The inward rotation of the C-terminal domain is indicated by the red curved arrow in **(B)**.

**FIGURE 9:**
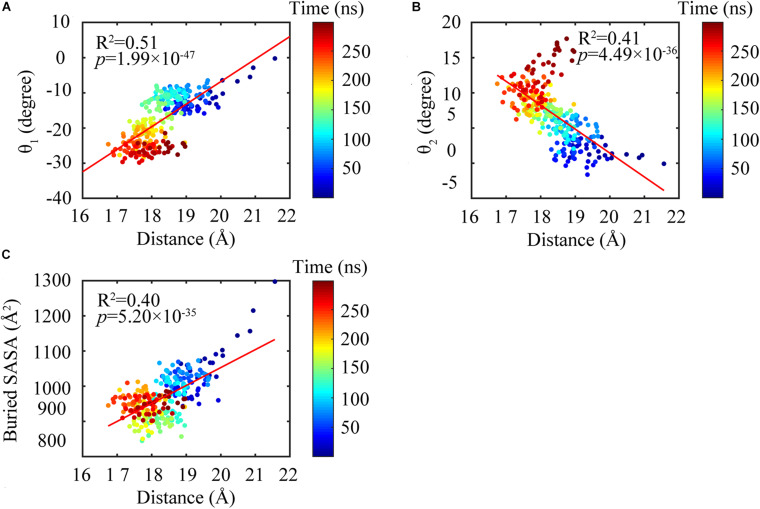
Subunit conformations were highly correlated with the dimeric conformations. Highlighting the changing of the distance between the N- and C-terminal domains were highly correlated with bending angles θ_1_ **(A)** and θ_2_ **(B)** and buried SASA **(C)**.

We found that the T-to-R conformation transitions are correlated with the dimeric conformations. The average subunit RMSDs revealed that the GDP-bound subunits exhibit larger deviations than that of the GTP-bound state (Figure 6 and Supplementary Figure 9). Further analysis of the distribution of the mean inter-domain distance revealed that the GTP-bound T conformation and the GDP-bound R conformation are well defined and separated by a clear inter-domain distance about 2.5 Å (Figure 7). The conformational switch from T-to-R involves an inward rotation of ∼19° (calculated by DynDom (Hayward and Berendsen, 1998)) of the C-terminal domain (Figure 8B). In addition, we monitored the T-to-R transitions of all FtsZ subunits, including bottom, middle and top ones, in GTP-bound and GDP-bound trajectories individually (Supplementary Figure 4) and parallelly (Supplementary Figure 10). Although all the three subunits in GDP-bound state have the tendency to finally transition to R conformations, we found that the middle subunit experiences a period of relatively higher inter-domain distance for nearly 150 ns (Supplementary Figure 10B, green line), implying that the internal structural dynamics are non-homogeneously distributed within a filament. The subunits at two ends are more dynamic than the middle one, which is stabilized by subunits above and below. Importantly, we observed that the T-to-R transitions in GDP-bound subunits exhibit considerable correlation with the twisting (θ_1_), bending (θ_2_) and open-closed motions between subunits (Figure 9). This indicated that the T-to-R conformation transitions are correlated with the dimeric conformations.

### Nucleotide Regulated Flexibility in FtsZ

**FIGURE 10:**
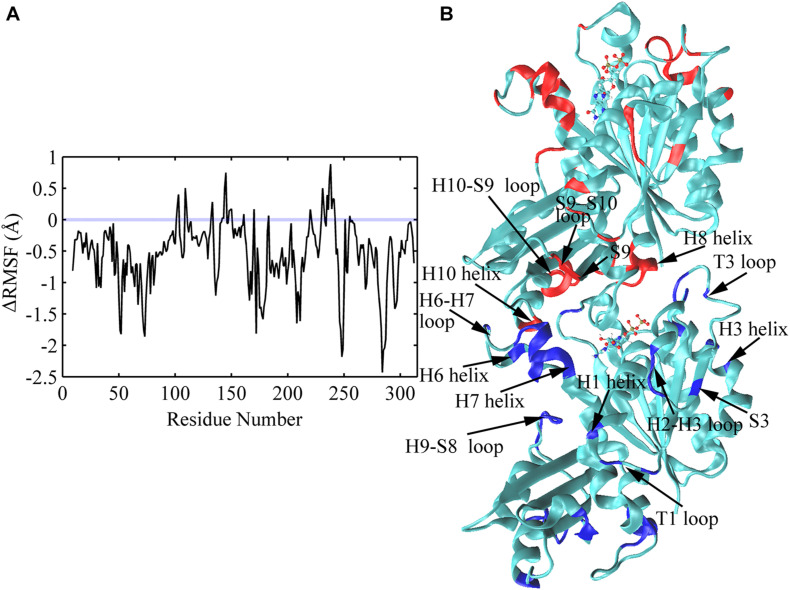
Comparison of subunit RMSFs between GTP- and GDP-bound states. **(A)** Difference in RMSF values of each amino acid between GTP- and GDP-bound states averaged over the full trajectories. Positive values denote higher flexibility in the GTP-bound state, and negative values denote higher flexibility in the GDP-bound state. Amino acids exhibiting the largest nucleotide dependent differences in flexibility (> 1.0 Å) were discussed in the main text, and highlighted with blue and red colors in panel **(B)** in a dimeric snapshot obtained from GDP-bound trajectories.

The average root mean square fluctuations (RMSFs) of the C_α_ atoms of FtsZ subunit are shown in Figure 10 and Supplementary Figure 11. GTP hydrolysis markedly changes the subunit flexibility. The GDP-bound state has larger RMSFs in both N- and C-terminal domains than that of the GTP-bound state (Supplementary Figure 11). Similar with the previous MD simulations (Hsin et al., 2012), GTP hydrolysis induced the higher flexibilities of the covering residues around the dimerization interface, i.e., the interface between the C-terminal domain of the top subunit and the N-terminal domain of the bottom subunit, especially the residues near the bound nucleotide (Figure 10). Surprisingly, there are some residues exhibiting higher flexibility in the regions far from the nucleotide binding site and the interface, including H1 (Q30), T1(L32, G34), H2-S3 loop (S50-A52), S3 (K55), H3 (D81) and H9-S8 loop (L246-S250) (Figure 10). This implies a possible long-range allosteric effect of the nucleotides on FtsZ’s flexibility and dynamics. The fluctuation in both H2/H2-S3 regions (A44, S50-A52, around the switch I region (Leung et al., 2004)) and T3 loop (R64, G67-A70, in switch II region) increases upon GTP hydrolysis, indicating that nucleotide binding and hydrolysis is coupled to the perturbation of the secondary structural fluctuations. In addition, the dynamic changes at loop regions are in coordination with structural changes of the whole protein (Skliros et al., 2012; Papaleo et al., 2016). Particularly, our results show that the regions with higher flexibility mainly distribute around loops (Figure 10). Interestingly, both the bottom region of the top subunit, including S9 sheet (I295), S9-S10 loop (D296-S298) and H10 helix (G268-F270), and the top region of the bottom subunit associated with nucleotide binding and hydrolysis, including T5 loop, H6-H7 loop, H6 and the tip of H7 helices, exhibit higher flexibility. Thus, the flexibility change induced by GTP hydrolysis to GDP in the bottom subunit is presumably transmitted to the top subunit through direct interactions. Such a long-range allosteric effect of nucleotide is captured by our simulation.

**FIGURE 11:**
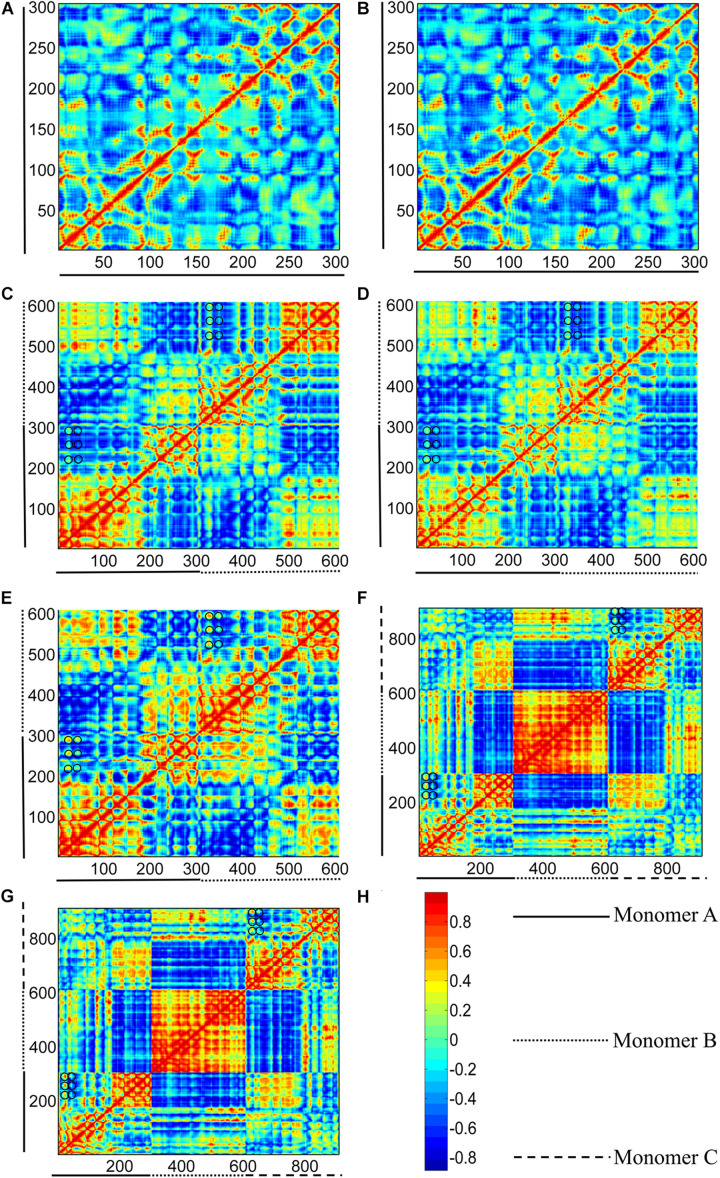
Correlation maps between residue fluctuations calculated from GTP- and GDP-bound structure I (see Figure 1). **(A,B)** subunits in GTP- and GDP-bound structure I, respectively. **(C–E)** bottom (middle-bottom) and top (top-middle) dimers in GTP-bound structures I, and dimer in GDP-bound structure I. **(F,G)** trimers for GTP- and GDP-bound structure I. The two axes refer to residue indices. As shown in the color bar **(H)**, blue and red regions correspond to negatively (opposite-direction) and positively (same-direction) correlated motions, respectively. Important residues were highlight with black circles.

### Insights Into the Subunit Structural Switch

Detailed information about the rearrangement of the intra-subunit contacts during the structural movement is shown in Supplementary Table 1 and Supplementary Figure 11. The difference of the intra-subunit contacts between GTP- and GDP-bound states reveals subtle intramolecular rearrangements, and indicates that the inter-domain’s communication is indeed mediated by the central regions. During the T-to-R transitions, the contacts between H5 helix (R152, E153) and S7 sheet (G219, T220), and between H6 helix (R165, L166) and H9-S8 loop (P245, L246) were missing (Supplementary Table 1 black and Supplementary Figure 11B). The switching of these interactions can play paramount roles in the T-to-R transitions. Significant changes of the inter-domain interactions mediated by the central regions (Supplementary Figure 11), including H7 helix (Supplementary Table 1, blue), T7 loop (Supplementary Table 1, red) and H8 helix (Supplementary Table 1, green), suggest a dominant role of the central regions in inter-domain’s communication. Especially, key contacts made by the T7 loop (G202-N205) with the S9 sheet (F291-T293, I295) are present in the GTP-bound open-cleft subunit, whereas all of these interactions are lost in the GDP-bound closed-cleft subunit. Some other residue contacts between H7 helix (V186, G190) and the S7 (M223) and S10 sheets (T306) of the C-terminal domain formed during the T-to-R transitions, accompanying with the disruption of contacts between H7 helix (Q192, G193) and the S7 (M223, G224), S10 sheets (V307) of the C-terminal domain. Meanwhile, subtle structural rearrangements were captured in the two simulation (Supplementary Table 1, blue and italic), i.e., initially the bottom part of H7 helix (L196, L197) contacts with S10 sheet (T306, I308) in the GTP-bound state, whereas this region eventually forms contacts with S9 sheet (G292, V294) in the GDP-bound state. It should be noted that most of the observed residue contacts among H7 helix, and S7 and S10 sheets are participated in the regulation of PC190723 (an SaFtsZ inhibitor) on the inter-domain motions (Matsui et al., 2012). In addition, two conserved contacts (Supplementary Table 1, blue and bold) between H7 helix (D184, V191) and the H1 helix (M27), T1 loop (V35) of the N-terminal domain are observed during the T-to-R transitions which are consistent with previous studies (Martín-Galiano et al., 2010; Fujita et al., 2017; Wagstaff et al., 2017).

### Comparison of Our Results With Previously Published Models

As shown in Supplementary Figure 4, the top end subunit (blue line) can switch to R conformation in either GTP-bound or GDP-bound state. We have shown that the top interface tends to be easier to open than that of the bottom interface (Figures 2, 3 and Supplementary Figures 3, 6), thus the top end subunit tends to switch to R and rapidly dissociates. This agrees with previous experimental and theoretical studies (Du et al., 2018; Corbin and Erickson, 2020). Importantly, the middle subunit and the bottom end subunit can be seen as a nucleation related dimer. Interestingly, there are three main subcategories of the dimer species, e.g., T-T (middle-bottom, Supplementary Figure 4, ellipse c) dimer in the GTP state, R-R (middle-bottom, Supplementary Figure 4, ellipse b) and T-R (middle-bottom, Supplementary Figure 4, ellipse d) dimers in the GDP state. A close look at the detailed order of switching of each subunit revealed that the disassembly speed of the T-to-R transition for GDP-bound middle subunit (Supplementary Figure 4, green line) is slower than that of the bottom end subunit (Supplementary Figure 4, red line). Dajkovic et al. proposed a related model in which two R subunits switch to T and then associate to make a T-T dimer (Dajkovic et al., 2008). This will lead to a subcategory dimer T-T (top-middle) in one GTP-bound simulation while in the disassembly end (Supplementary Figure 4, ellipse a). In agreement with Corbin and Erickson (Corbin and Erickson, 2020), the pathway is equivalent in an equilibrium situation and may represent a rare species. In addition, Miraldi et al. had suggested the existence of two possibilities of unstable intermediate dimer, T-R (top-bottom) or R-T (top-bottom) during the process to forming of a T-T dimer from two R subunits (Miraldi et al., 2008). Fujita et al. suggested that there exists a structural equilibrium between filaments in T and R states (Fujita et al., 2017). This would lead to a subcategory dimer R-R (middle-bottom, Supplementary Figure 4, ellipse b) in two GDP-bound simulations. However, Corbin and Erickson suggested that the structural equilibrium does not exist in solution but occurs upon crystallization (Corbin and Erickson, 2020). These seemingly contradictory findings raised an obvious question: which nucleation model is possible? Our simulation results revealed a subcategory dimer T-R (middle-bottom, Supplementary Figure 4B, ellipse d) during all GDP-bound simulations (Supplementary Figure 4A, red and blue lines) in the assembly end. Recently Corbin and Erickson had suggested that nucleation occurs when a T subunit binds an R subunit which then switches to T, resulting in a T-T dimer (Corbin and Erickson, 2020). This agrees well with our simulations results. They proposed two possible pathways following the initial association of an R subunit at the bottom end: it either switches to T and remains tightly bound, or dissociates before switching. In our results, we found that the bottom end subunit shows a nucleotide-regulated transition: R tends in GDP-bound state and T tends in GTP-bound state. Taken together, we suggested a more feasible nucleation pathway: one R and one T subunits associate to form a T-R (top-bottom) dimer, which then switches to a T-T dimer.

### Analysis of Domain Motions

To remove the border effects that are induced in MD simulations, we calculated the correlation between residue fluctuations based on ANM method. As shown in Figures 11A,B, the cross-correlation maps based on fluctuation calculations for GTP- and GDP-bound monomeric structures cannot reveal the distinctive regions indicative of correlated motions. By contrast, in the dimeric structures, the residues within the N- and C-terminal subdomains of each subunit have positive correlations within their own regions (Figures 11C–E), indicating that the N- and C-terminal subdomains retain their structures during the inter-domain motions. Meanwhile, large negative correlations exist between the two subdomains of each subunit, reflecting that each subunit of dimer can individually undergo a T-R transition. Further comparison of the cross-correlations between GTP- (Figures 11C,D) and GDP-bound (Figure 11E) states revealed that the relative weak correlations between some residues of different domains in the GTP-bound dimer (Figures 11C,D, black circles), become positively stronger in GDP-bound dimer (Figure 11E, black circles). These residue pairs mainly distribute between the regions of T1 and T2 loops in the N-terminal subdomains, and the regions of S7/S8/S9 sheets and S7-H9/S8-H10/S9-S10 loops in the C-terminal subdomains. During the T-to-R transitions, more interactions form between the residues in the N-terminal subdomain and those in the C-terminal subdomain in the front of each subunit. In addition, the relative motions between the N- and C-terminal subdomains of the bottom subunit are negatively correlated with their counterparts in the top subunit. This indicates that each subunit exhibits symmetrical, opposite direction motions with respect to their dimer interface.

Such a T-R transition also exists in the two end subunits of a trimer (Figures 11F,G). It is worth noting that the middle subunit of a trimer is positively correlated, indicating that its motion is relatively rigid. This can be attributed to the fact that the middle subunit is stabilized by the adjacent subunits. We further tested such correlated motions in a tetramer and a pentamer (Supplementary Figure 13). Interestingly, we observed that the correlated motions in the two end subunits are gradually reduced with more subunits in a filament, (Supplementary Figure 13). The results based on ANM analysis suggested that a trimer can access several subcategories of dimer species that have been observed in MD simulations. Thus, strong inter-domain and inter-subunit cooperative motions are observed in the dimer and trimer structures. While in the subunits, residues are only correlated with nearby residues. Local connectivity may be the driving force for subunit motion, whereas long-range effects play a dominant role in the allosteric signal transmission and conformational couplings in polymers (Keskin et al., 2002; Atilgan et al., 2007). The results of ANM calculation suggest that the enhanced assembly cooperativity is encoded in the topological features of FtsZ polymer.

## Discussion

In this work, we performed molecular dynamics simulations on the GTP- or GDP-bound MtbFtsZ trimers. Different interface configurations have been observed in previous MD simulations, i.e., multiple intermediate conformations between the closed and open interfaces coexist along the GDP-bound filament rather than that of the GTP-bound ones (Ramírez-Aportela et al., 2014). Similarly, we observed that the GDP-bound trimer exhibits a higher interfacial variability. On the other hand, our simulation showed that the closed (bottom) and open (top) interfaces can also coexist within the GTP-bound trimer, which is different from previous studies. Such a discrepancy indicates that the top end of a filament is highly dynamic, which is prerequisite for treadmilling. Thus, our results suggested that the top and bottom ends of FtsZ filament are the kinetic minus and plus ends, respectively, confirming the previous experimental studies (Osawa and Erickson, 2005; Redick et al., 2005; Du et al., 2018). Notably, one of the five trajectories showed that both top and bottom interfaces are closed (Supplementary Figure 3).

The regulation of nucleotide state on the bending flexibility of inter-subunit interfaces were also studied by calculating three rotational angles between subunits (θ_1_, θ_2_, θ_3_, Figure 5). The preferred bending directions are on θ_1_ and θ_2_. The importance of the preferred bending directions around θ_1_ and θ_2_ on generating filament curvature and force have been highlighted (Hsin et al., 2012; González De Prado Salas et al., 2014; Ramírez-Aportela et al., 2014). The mean values of θ_1_ in GTP- and GDP-bound states are ∼−25° and ∼−20°, respectively. The decomposition of θ_1_ based on the four identified interfaces (GTP-IF1, GDP-IF1, GTP-IF2 and GDP-IF2) shows the ability of the FtsZ filament to access a large range of θ_1_ from ∼0° to ∼35°, indicating a substantial plasticity of the FtsZ filament. The combination of the bending and twisting motions between FtsZ subunits would lead to the assembly of helical FtsZ filaments. Arumugam et al. (Arumugam et al., 2012) observed that *E. coli* FtsZ filament can form helices with a pitch of about 150 nm, leading to a twist of ∼4.8° per subunit (∼4 nm). This is compatible with those of GTP-IF1 (−6.1°) and GDP-IF1 (−9.4°) interfaces. By contrast, the crystal structures obtained by Li et al. (2013) and Guan et al. (2018) showed that the MtbFtsZ filament forms helices with six subunits per turn, a small pitch of 13 nm, leading to a twist of ∼30° per subunit. Meanwhile, an EM study showed a highly curved PaFtsZ protofilament with a pitch of about 19 nm, leading to a twist of ∼34.3° per subunit. These are compatible with those of GTP-IF2 (−35.7°) and GDP-IF2 (−33.1°) interfaces. To clearly show the twisting directions, we calculated the twisting angles (θ_1_) in the structures determined in different crystal forms. By comparing our simulation results with the structures determined in different crystal forms (Supplementary Figure 14), we had two findings. First, FtsZ can twist in two opposite directions, supporting the earlier experimental evidence (Ramirez-Diaz et al., 2018). An inherent helical character of the filaments with more than one direction of curvature have been observed by both theoretical (González De Prado Salas et al., 2014) and experimental studies (Arumugam et al., 2012; Márquez et al., 2019). Second, the dimers with the smaller twisting angles are in tightly associated conformations, while larger twisting angles result in the loosely associated conformations. Meanwhile, as the pitch size of a helical filament is directly related to the size of the twisting angle, we suggest that GTP-bound FtsZ can form less twisted (<∼6°) filament with large-sized pitches, while GTP hydrolysis promotes FtsZ to form highly twisted (>∼6°) filament with small-sized pitches. Several lines of experimental evidence from *in vitro* studies (Arumugam et al., 2012; Mateos-Gil et al., 2019; Márquez et al., 2019) suggest that FtsZ filaments are twisted, which is functionally important, particularly considering the also frequently overlooked fact that filaments are associated to the membrane through a flexible linker. By contrast, Turner et al. (2012) suggested that the FtsZ fibers have no intrinsic global or local curvatures. Thus, exactly how much force exerted by FtsZ on the inner side of the cell membrane during cell division is due to the formation of helical structures remains elusive.

Both experimental (Artola et al., 2017) and structural studies (Wagstaff et al., 2017) proposed that the assembly switch of FtsZ involves inter-domain motions, i.e., opening clefts between subdomains, which are allosterically coupled to the formation of the longitudinal inter-subunit interfaces along the filament. Our results validated such a scenario and indicated that polymerization-associated assembly switch is driven by coupled motions between subdomains as well as the motion between subunits. Moreover, cross-correlation maps revealed that strong inter-domain and inter-subunit coupled motions are observed in both the dimer and trimer, while the correlation within subunit is very limited. These findings are in line with previously proposed views that polymerization enhances the assembly cooperativity (Wagstaff et al., 2017).

By analyzing the independent simulation trajectories, three subcategories of the dimer species, e.g., T-T (middle-bottom) dimer in GTP state, R-R (middle-bottom) and T-R (middle-bottom) dimers in GDP state, were observed. It should be noted that the MD simulations with trimers may be influenced by border effects. However, both the ANM results (without the border effects) and the convergence between our MD simulations and previous models lead us to believe that the results of our MD simulations sampled different representative conformations of FtsZ, instead of the subject to border effects. Our data lead us to propose a model for FtsZ assembly-disassembly mechanism. In cooperative polymerization, assembly initiates in a nucleation step, followed by an elongation step (Oliva et al., 2004; Chen and Erickson, 2009). During nucleation stage, one GTP-bound R subunit and one GTP-bound T subunit associate to form a T-R (top-bottom) dimer which then forms a GTP-bound T-T dimer. Once the dimer nucleus comes into shape, the additional subunits may use similar pathways as nucleation to elongate from the bottom end. Simultaneously, polymerization-associated nucleotide hydrolysis increases the flexibility of subunits and causes filament depolymerization from the top end.

Taken together, we have four important findings. First, we observed both homogeneous and heterogeneous distributions of T and R conformations in FtsZ dimers of trimers, including T-T, T-R and R-R dimers. Second, the top end subunit of a filament tends to undergo T-to-R transitions in both GTP- and GDP-bound states. Third, FtsZ filament exhibits noticeable amounts of twisting (θ_1_), ranging from ∼0° to ∼35°, in agreement with previous experimental data, indicating a substantial helicity of the FtsZ filament. Fourth, ANM analysis revealed a polymerization enhanced assembly cooperativity. It should be noted that our results do not exclude other possible assembly dynamics, considering FtsZ as ‘soft’ filaments that can also associate with each other and with other partners.

## Data Availability Statement

The raw data supporting the conclusions of this article will be made available by the authors, without undue reservation.

## Author Contributions

SY, DL, and JL conceived and designed this project. DL performed the simulations and analyzed the data. All authors participated in the data analysis and manuscript.

## Conflict of Interest

The authors declare that the research was conducted in the absence of any commercial or financial relationships that could be construed as a potential conflict of interest.

## Footnotes

1 http://swissmodel.expasy.org/

2 http://anm.csb.pitt.edu/
