# Supplementary material for: The Assembly Switch Mechanism of FtsZ Filament Revealed by All-Atom Molecular Dynamics Simulations and Coarse-Grained Models
Source: Front Microbiol. 2021 Mar 30;12:639883. doi: 10.3389/fmicb.2021.639883 (PMC8042166; doi:10.3389/fmicb.2021.639883)
Supplement: Supplementary file 1 [file Data_Sheet_1.pdf]

Legends of supplementary materials

Supplementary Figure S1. T to R transitions of GTP-bound monomers in the 300 ns simulation. The time evolution of the distance between the centers of mass of the end of the N-terminal (residues 9-50) and the S9 sheet strand (residues 292-296) from the C-terminal domain during the MD simulation is shown. Blue line: top monomer of a trimer. Green line: middle monomer of trimer. Red line: bottom monomer of a trimer. The initial distance is about 32 Å^2.

Supplementary Figure S2. Free energy landscape (FEL) plot of GTP- (A) and GDP-bound (B) subunits during whole production dynamics. PC1 and PC2 are the first and second singular vectors obtained from SVD analysis, respectively. The z axis corresponds to free energy (k_BT).

Supplementary Figure S3. Changes of buried SASA account for dimers of trimers in every independent trajectory. Blue line: top interface. Red line: bottom interface. Black dashed line: original buried SASA (about 1200 Å^2). Rectangle: first 100 ns simulations, which indicates that the open speed of the top interface tend to be earlier than that of the bottom interface. The interfaces with values of buried SASA bigger than 1100 Å^2 and smaller than 1000 Å^2 are identified as the open and closed interfaces, respectively.

Supplementary Figure S4. T-to-R transitions of subunits in every independent trajectory. (A) The time evolution of the distance between the centers of mass of the end of the N-terminal (residues 9-50) and the S9 sheet strand (residues 292-296) from the C-terminal domain during the MD simulation is shown. Blue line: top monomer of a trimer. Green line: middle monomer of trimer. Red line: bottom monomer of a trimer. Black dashed line: the initial inter-domain's distance (about 32 Å). The inter-domain's distance for the fully R conformation is about 24 Å in MtbFtsZ (PDB ID: 5ZUE). **(B)** The final conformations correspond to independent runs in **(A)** were shown (same as structures in **FIGURE 1**). The main subcategories of the dimer species were indicated by ellipses.

Supplementary Figure S5. Comparison of the subunits within the final simulated structures shown in **FIGURE 1** with the initial subunit in T state (gray) and the MtbFtsZ (PDB ID: 5ZUE) subunit in R state (orange). In each panel, the bottom (blue), middle (yellow) and top (red) subunits are shown in the same color as those in **FIGURE 1**. (A) Subunits in structure I in GTP state. (B) Subunits in structure II in GTP state. (C) Subunits in structure I in GDP state. (D) Subunits in structure II in GDP state. (E) Subunits in structure III in GDP state. The initial inter-domain's distance is about 32 Å. The inter-domain's distance for the fully R conformation is about 24 Å in MtbFtsZ (PDB ID: 5ZUE). The inter-domain's distance for the final subunit are indicated around structures. It should be noted that the calculated inter-domain distances in R conformation are between ~26.8 Å (closer to the fully R conformation MtbFtsZ (PDB ID: 5ZUE, about 24 Å)) and ~29.5 Å. Although the

inter-domain distances of three subunits (the two end subunits in GDP-bound structure III and the top end subunit in GTP-bound structure II) are ~29.5 Å (closer to T), we identified them as intermediate structures with R features during the T-to-R transitions.

Supplementary Figure S6. Changes of buried SASA account for interfaces of trimers. (A) Measurement of buried SASA for the top (blue) and the bottom (red) interfaces of GTP-bound trimers. **(B)** Measurement of buried SASA for the top (blue) and the bottom (red) interfaces of GDP-bound trimers. The interfaces start from the same value about 1200 Å^2. In all the cases, the buried SASAs are calculated by averaging all the GTP-bound (two runs) or GDP-bound (three runs) trajectories.

Supplementary Figure S7. Measurement of the relative rotations between subunits in the GTP- (blue) and GDP-bound (red) trimers. (A) Time evolution of θ_1, which corresponds to the averaged "twisting" motions for the top and bottom dimer interfaces over all trajectories. **(B)** Time evolution of θ_2, which corresponds to the averaged "bending" motions for the top and bottom dimer interfaces over all trajectories. **(C)** Time evolution of θ_3, which describs the averaged bending motions for the top and bottom dimer interfaces orthogonal to θ_2 over all trajectories.

Supplementary Figure S8. Distribution of the twisting angle θ_1 between subunits for different types of interfaces. GTP-IF1: blue. GTP-IF2: yellow. GDP-IF1: red. GDP-IF2: green. The highest probability values of corresponding interfaces are indicated with dashed lines and its values are shown above. The angles are from a starting zero for initial dimer conformation.

Supplementary Figure S9. RMSDs of subunits evaluated along the trajectories. (A) RMSDs of every subunit within the GTP-bound trimers along trajectories. Color scheme: red for bottom end subunit, green for middle subunit, yellow for top end subunit, blue for averaged RMSD of all the three subunits. **(B)** RMSDs of each subunit within the GDP-bound trimers along trajectories. The color scheme is same to that of **(A)**. In all the cases, the RMSDs are calculated by averaging all the GTP-bound (two runs) or GDP-bound (three runs) trajectories.

Supplementary Figure S10. T-to-R transitions of monomers in GTP-bound (A) and GDP-bound (B) simulations. The distances between the centers of mass of the end of the N-terminal (residues 9-50) and the S9 sheet strand (residues 292-296) from the C-terminal domain are calculated. Color scheme: red for bottom end subunit, green for middle subunit, yellow for top end subunit, blue for averaged RMSD of all the three subunits. In all the cases, the distances are calculated by averaging all the GTP-bound (two runs) or GDP-bound (three runs) trajectories.

Supplementary Figure S11. Averaged RMSFs of the independently aligned subunits of the GTP- (red) and GDP-bound (blue) trimers.

Supplementary Figure S12. Evolution of the breaking (blue bond) and forming (orange bond) of the residue contacts within subunits during the inter-domain's open-to-closed motions. The structure was obtained from the final stable trajectories of the GDP-bound simulations. **(A-C)** represent the front, back and top views respectively. The central regions were highlighted with red color.

Supplementary Figure S13. Correlation maps between residue fluctuations in a tetramer (A) and a pentamer (B). The structures are generated by crystallographic symmetry operations based on the initial T subunits. Blue and red regions correspond to negatively (opposite-direction) and positively (same-direction) correlated motions, respectively.

Supplementary Figure S14. Comparison of the twisting of FtsZ dimers between the four identified dimers and the dimers determined in different crystal forms.

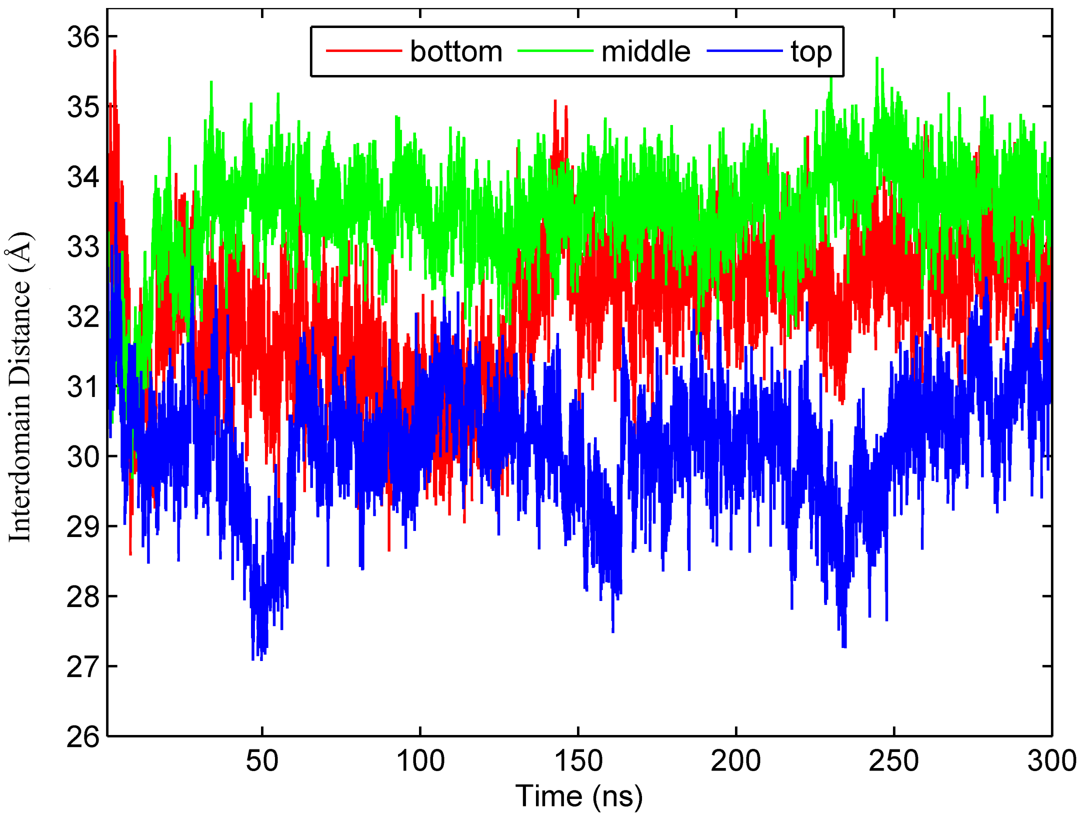

Supplementary Figure S1. T to R transitions of GTP-bound monomers in the 300 ns simulation. The time evolution of the distance between the centers of mass of the end of the N-terminal (residues 9-50) and the S9 sheet strand (residues 292-296) from the C-terminal domain during the MD simulation is shown. Blue line: top monomer of a trimer. Green line: middle monomer of trimer. Red line: bottom monomer of a trimer. The initial distance is about 32 $Å^2$.

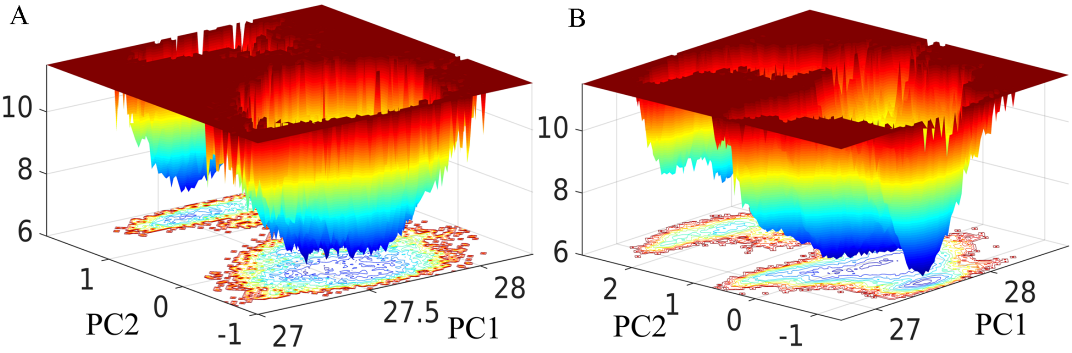

Supplementary Figure S2. Free energy landscape (FEL) plot of GTP- (A) and GDP-bound (B) subunits during whole production dynamics. PC1 and PC2 are the first and second singular vectors obtained from SVD analysis, respectively. The z axis corresponds to free energy (k_BT).

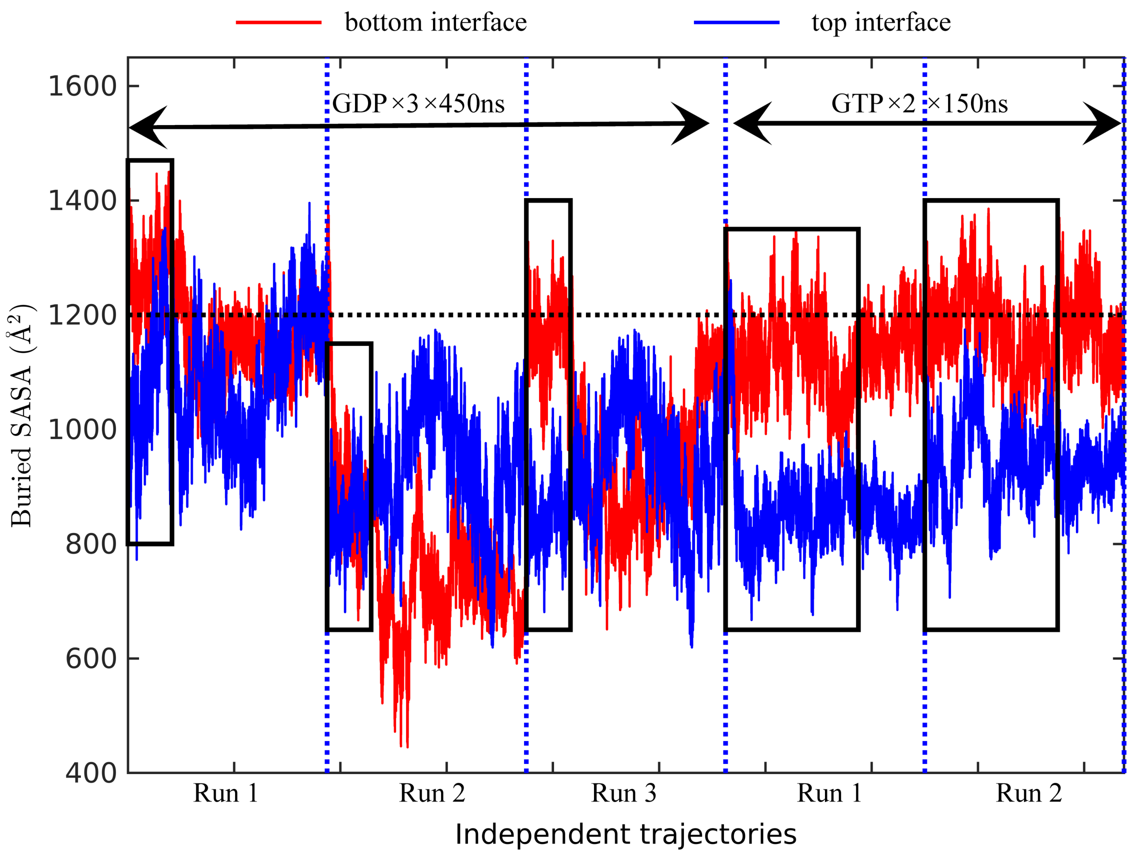

Supplementary Figure S3. Changes of buried SASA account for dimers of trimers in every independent trajectory. Blue line: top interface. Red line: bottom interface. Black dashed line: original buried SASA (about 1200 $Å^2$). Rectangle: first 100 ns simulations, which indicates that the open speed of the top interface tend to be earlier than that of the bottom interface. The interfaces with values of buried SASA bigger than 1100 $Å^2$ and smaller than 1000 $Å^2$ are identified as the open and closed interfaces, respectively.

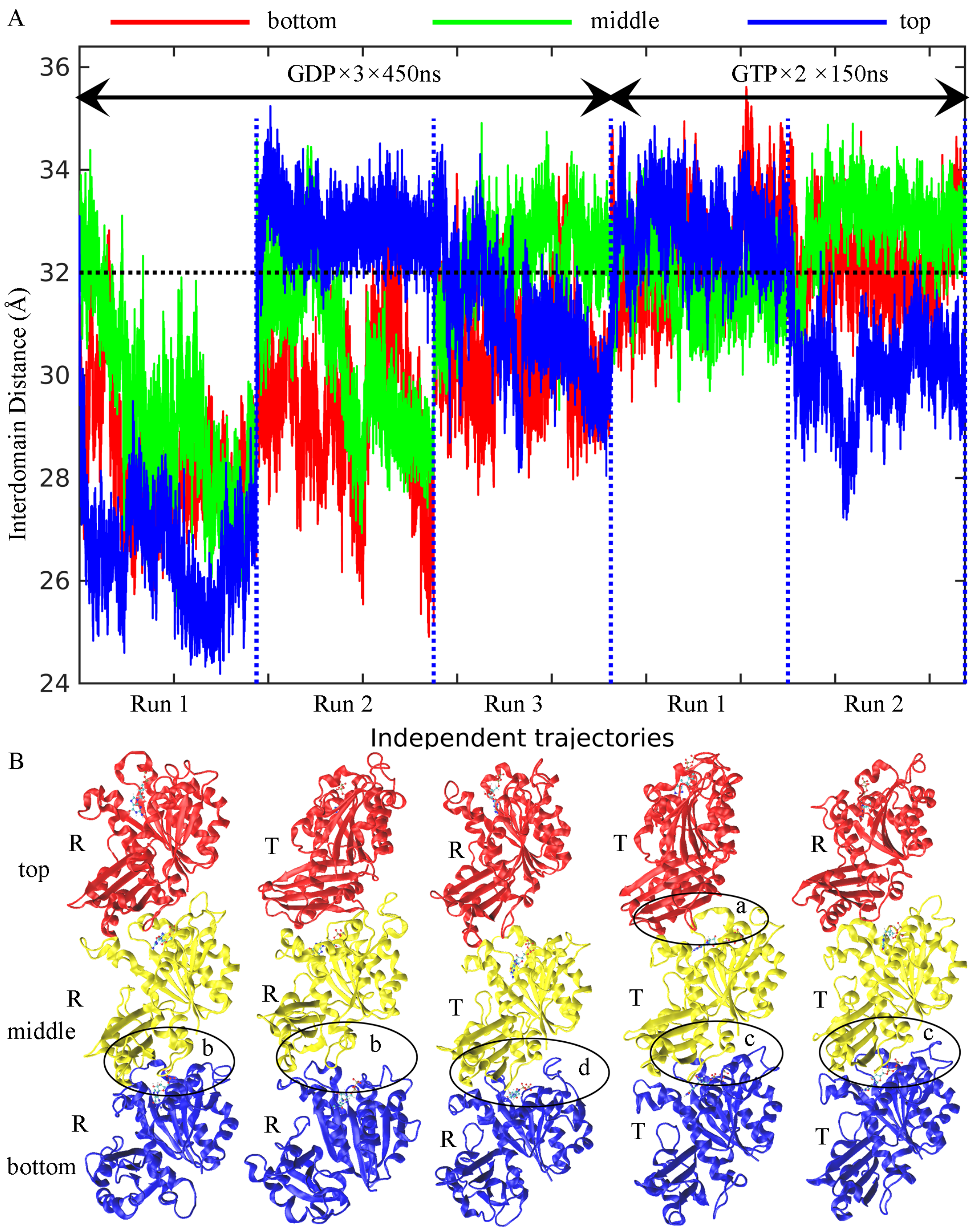

Supplementary Figure S4. T-to-R transitions of subunits in every independent trajectory. (A) The time evolution of the distance between the centers of mass of the end of the N-terminal (residues 9-50) and the S9 sheet strand (residues 292-296) from the C-terminal domain during the MD simulation is shown. Blue line: top monomer of a trimer. Green line: middle monomer of trimer. Red line: bottom monomer of a trimer. Black dashed line: the initial inter-domain's distance (about 32 Å). The inter-domain's distance for the fully R conformation is about 24 Å in MtbFtsZ (PDB ID: 5ZUE). **(B)** The final conformations correspond to independent runs in **(A)** were

shown (same as structures in **FIGURE 1**). The main subcategories of the dimer species were indicated by ellipses.

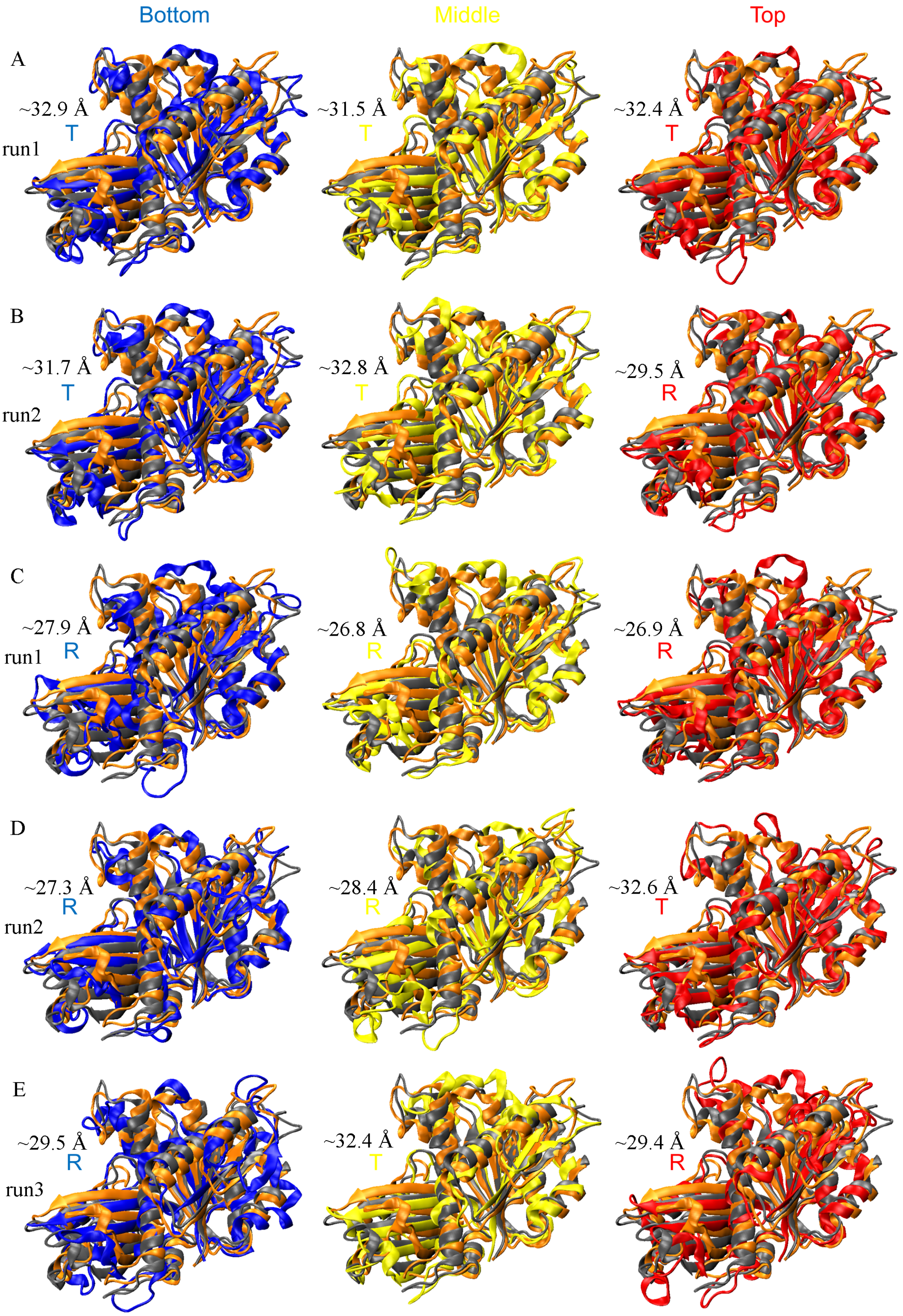

Supplementary Figure S5. Comparison of the subunits within the final simulated structures shown in **FIGURE 1** with the initial subunit in T state (gray) and the MtbFtsZ (PDB ID: 5ZUE) subunit in R state (orange). In each panel, the bottom (blue), middle (yellow) and top (red) subunits are shown in the same color as those in

FIGURE 1. (A) Subunits in structure I in GTP state. (B) Subunits in structure II in GTP state. (C) Subunits in structure I in GDP state. (D) Subunits in structure II in GDP state. (E) Subunits in structure III in GDP state. The initial inter-domain's distance is about 32 Å. The inter-domain's distance for the fully R conformation is about 24 Å in MtbFtsZ (PDB ID: 5ZUE). The inter-domain's distance for the final subunit are indicated around structures. It should be noted that the calculated inter-domain distances in R conformation are between ~26.8 Å (closer to the fully R conformation MtbFtsZ (PDB ID: 5ZUE, about 24 Å)) and ~29.5 Å. Although the inter-domain distances of three subunits (the two end subunits in GDP-bound structure III and the top end subunit in GTP-bound structure II) are ~29.5 Å (closer to T), we identified them as intermediate structures with R features during the T-to-R transitions.

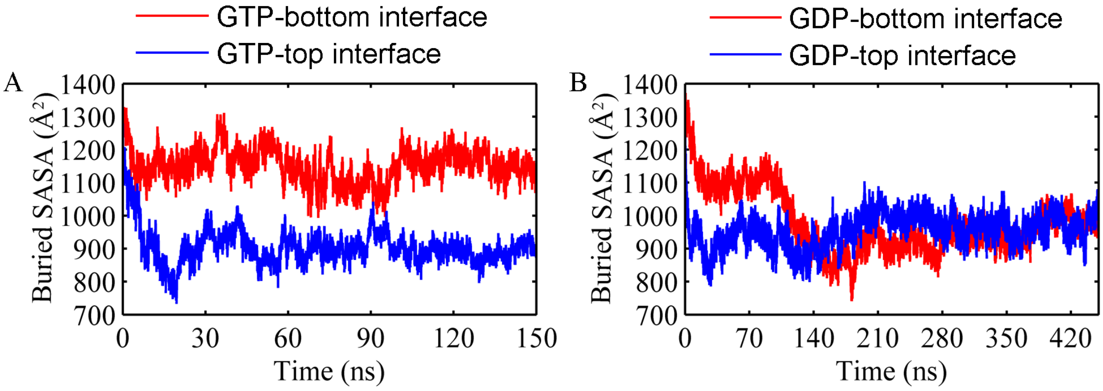

Supplementary Figure S6. Changes of buried SASA account for interfaces of trimers. (A) Measurement of buried SASA for the top (blue) and the bottom (red) interfaces of GTP-bound trimers. **(B)** Measurement of buried SASA for the top (blue) and the bottom (red) interfaces of GDP-bound trimers. The interfaces start from the same value about 1200 $Å^2$. In all the cases, the buried SASAs are calculated by averaging all the GTP-bound (two runs) or GDP-bound (three runs) trajectories.

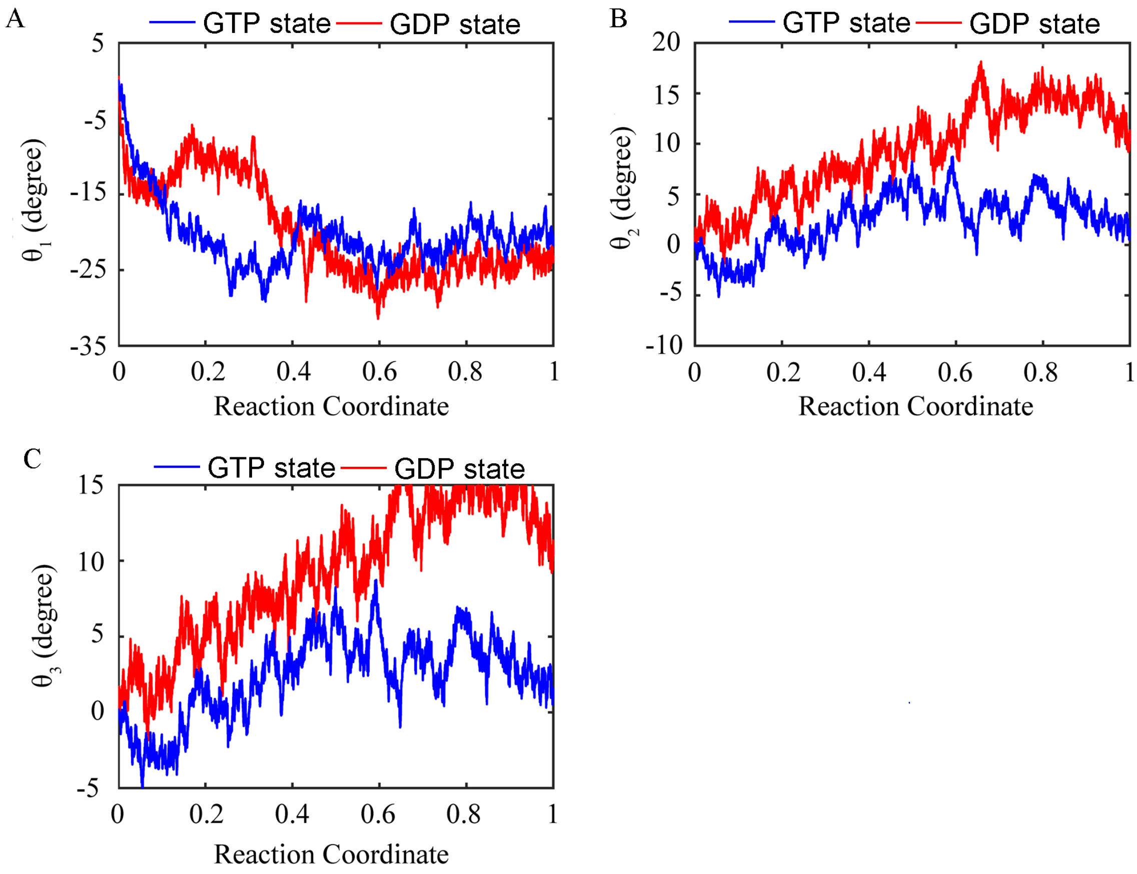

Supplementary Figure S7. Measurement of the relative rotations between subunits in the GTP- (blue) and GDP-bound (red) trimers. (A) Time evolution of θ_1, which corresponds to the averaged "twisting" motions for the top and bottom dimer interfaces over all trajectories. **(B)** Time evolution of θ_2, which corresponds to the averaged "bending" motions for the top and bottom dimer interfaces over all trajectories. **(C)** Time evolution of θ_3, which describs the averaged bending motions for the top and bottom dimer interfaces orthogonal to θ_2 over all trajectories.

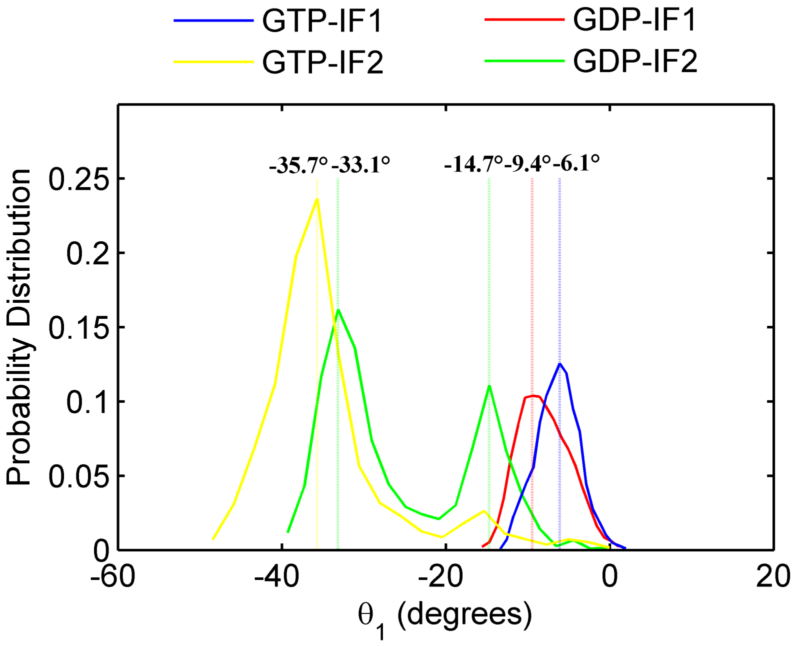

Supplementary Figure S8. Distribution of the twisting angle θ_1 between subunits for different types of interfaces. GTP-IF1: blue. GTP-IF2: yellow. GDP-IF1: red. GDP-IF2: green. The highest probability values of corresponding interfaces are indicated with dashed lines and its values are shown above. The angles are from a starting zero for initial dimer conformation.

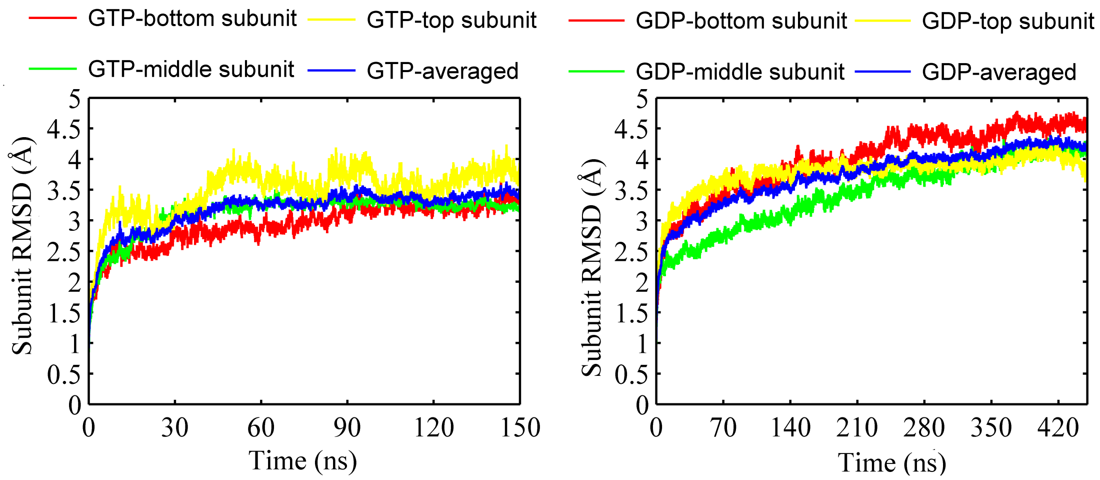

Supplementary Figure S9. RMSDs of subunits evaluated along the trajectories. (A) RMSDs of every subunit within the GTP-bound trimers along trajectories. Color scheme: red for bottom end subunit, green for middle subunit, yellow for top end subunit, blue for averaged RMSD of all the three subunits. **(B)** RMSDs of each subunit within the GDP-bound trimers along trajectories. The color scheme is same to that of **(A)**. In all the cases, the RMSDs are calculated by averaging all the GTP-bound (two runs) or GDP-bound (three runs) trajectories.

A

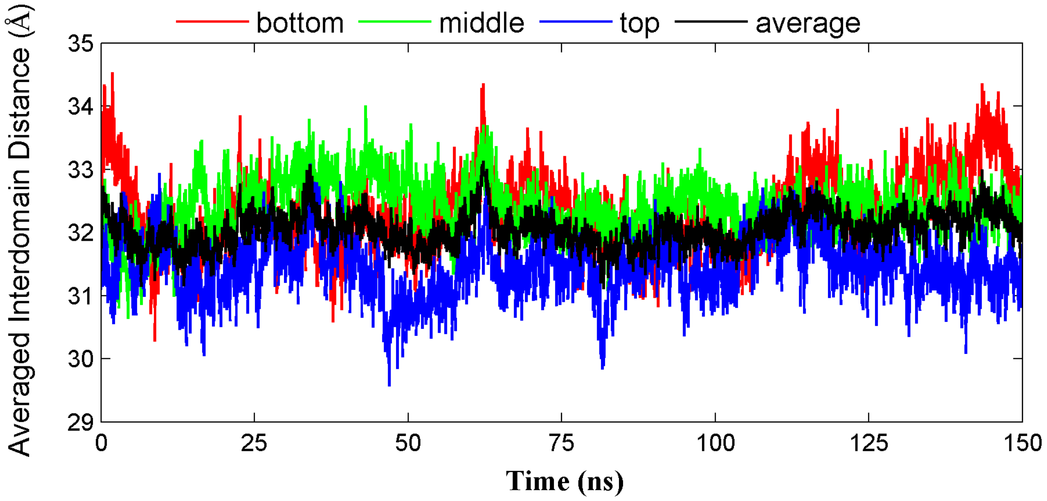

B

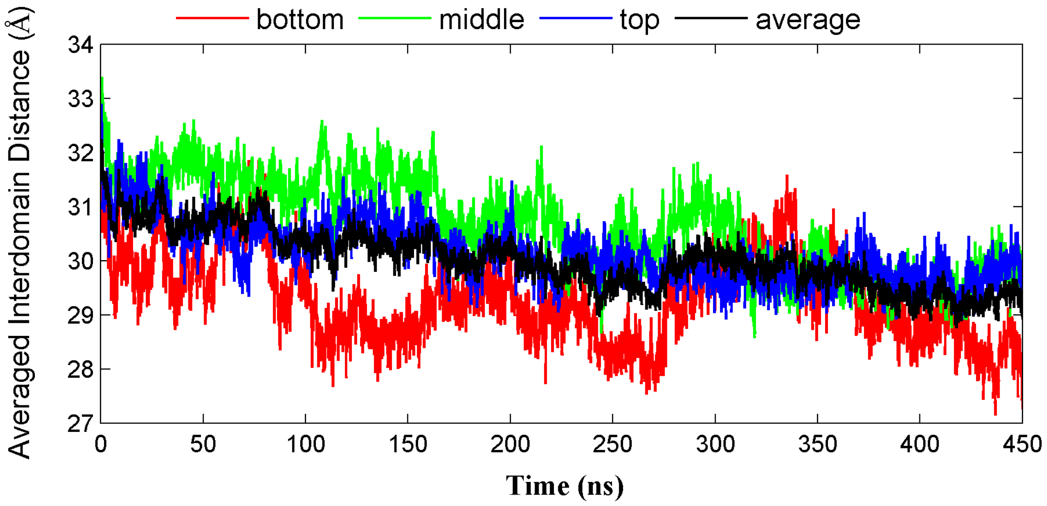

Supplementary Figure S10. T-to-R transitions of monomers in GTP-bound (A) and GDP-bound (B) simulations. The distances between the centers of mass of the end of the N-terminal (residues 9-50) and the S9 sheet strand (residues 292-296) from the C-terminal domain are calculated. Color scheme: red for bottom end subunit, green for middle subunit, yellow for top end subunit, blue for averaged RMSD of all the three subunits. In all the cases, the distances are calculated by averaging all the GTP-bound (two runs) or GDP-bound (three runs) trajectories.

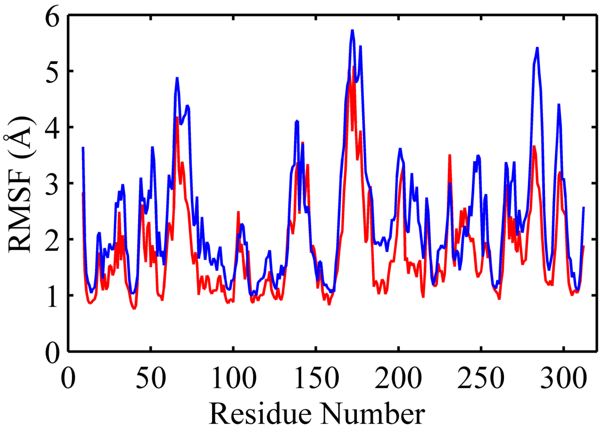

Supplementary Figure S11. Averaged RMSFs of the independently aligned subunits of the GTP- (red) and GDP-bound (blue) trimers.

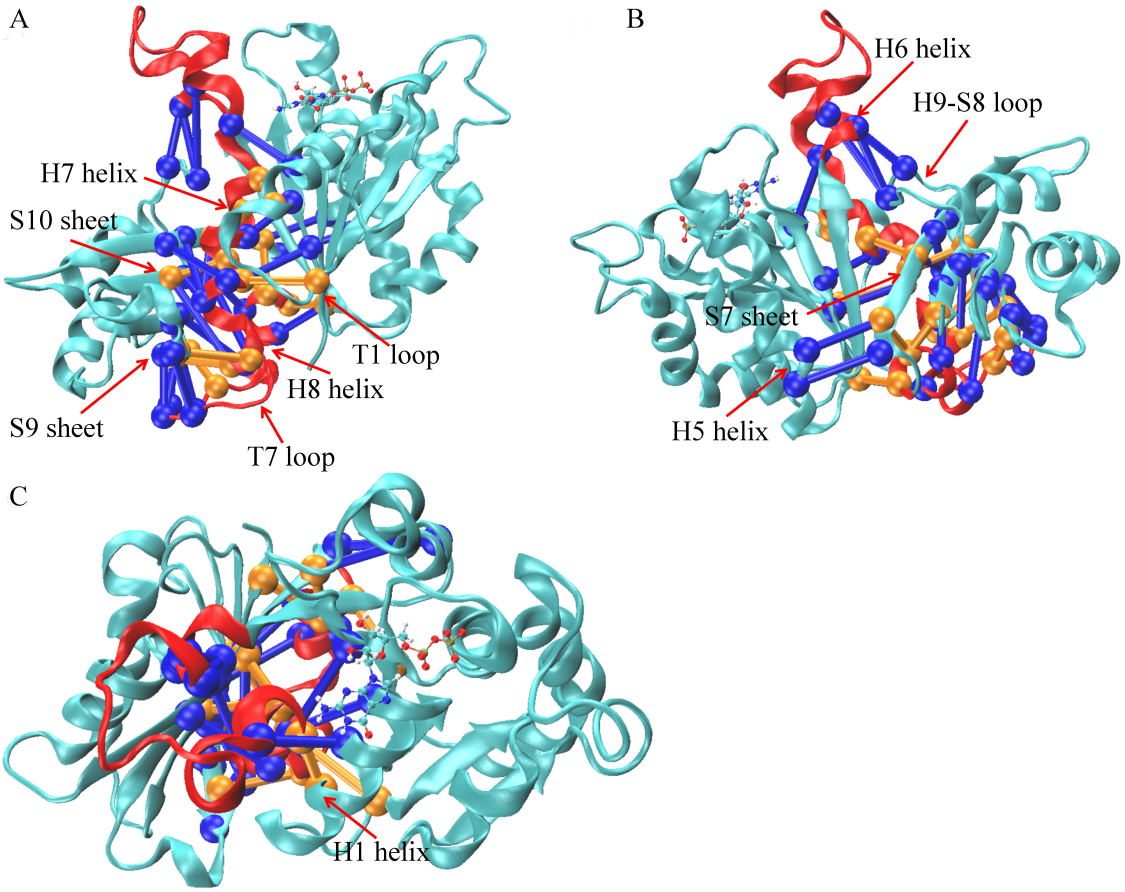

Supplementary Figure S12. Evolution of the breaking (blue bond) and forming (orange bond) of the residue contacts within subunits during the inter-domain's open-to-closed motions. The structure was obtained from the final stable trajectories of the GDP-bound simulations. **(A-C)** represent the front, back and top views respectively. The central regions were highlighted with red color.

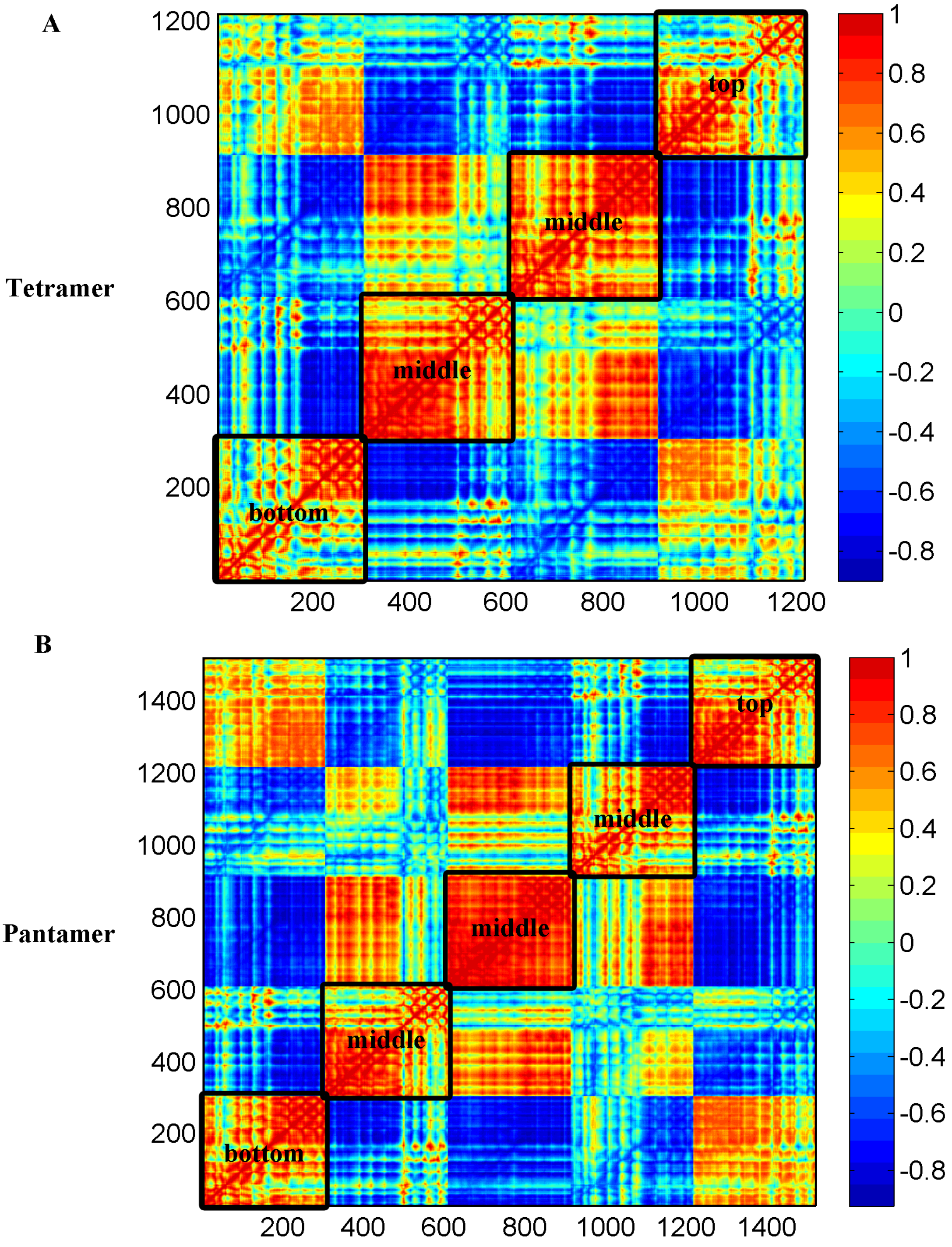

Supplementary Figure S13. Correlation maps between residue fluctuations in a tetramer (A) and a pentamer (B). The structures are generated by crystallographic symmetry operations based on the initial T subunits. Blue and red regions correspond to negatively (opposite-direction) and positively (same-direction) correlated motions, respectively.

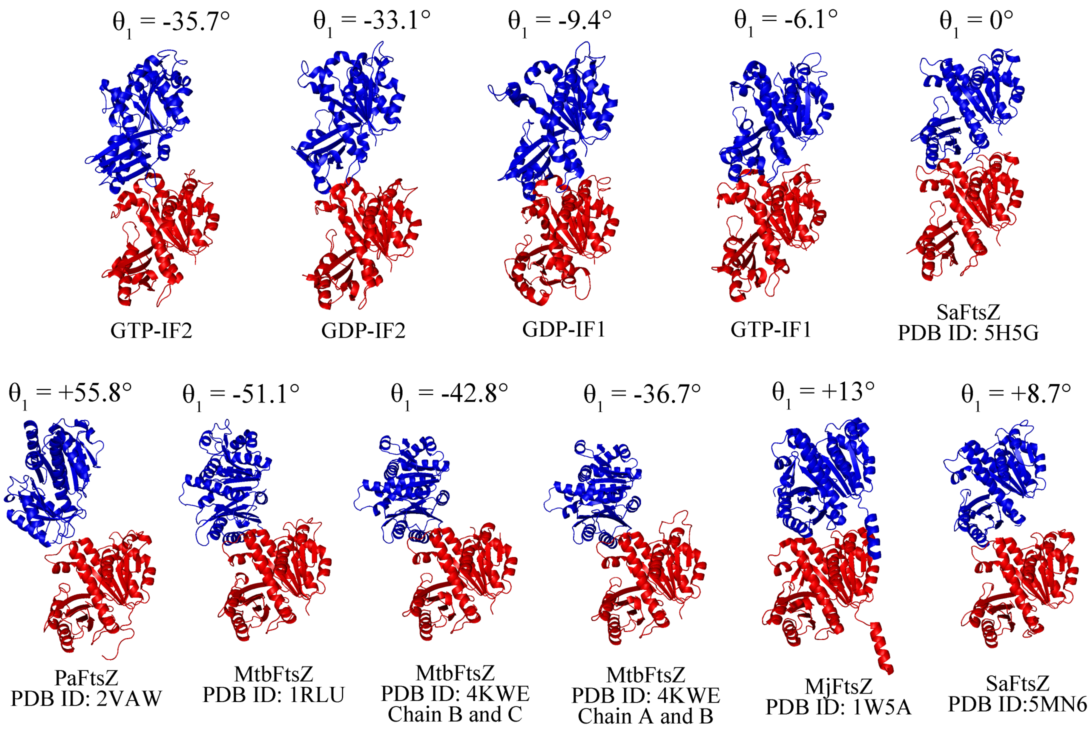

Supplementary Figure S14. Comparison of the twisting of FtsZ dimers between the four identified dimers and the dimers determined in different crystal forms.

Table S1. The difference of the contacts between the GTP-bound open-cleft and the GDP-bound closed-cleft subunits. Intra-subunit contacts were calculated between all pairs of residues in all subunits in each case using a cutoff distance of 8 Å. The contacts that presented for at least 50% of the simulation were shown. Positive values denote the contacts formed in the GDP-bound state, and negative values denote the contacts formed in the GTP-bound state.

Residue pairs		Percent(%)	Residue pairs		Percent(%)
A23	R181	-62.35%	G193	V307	-95.35%
**M27**	**D184**	**51.60%**	*D196*	*I308*	*-58.37%*
**V35**	**V191**	**50.39%**	*L196*	*V294*	*57.64%*
F97	V191	-64.77%	*L197*	*T306*	*-66.19%*
L124	K212	-58.15%	*L197*	*G292*	*58.74%*
L124	S216	63.34%	G202	G292	-50.53%
V128	L187	-99.61%	G202	I295	-58.73%
**R152**	**T220**	**-68.25%**	L203	G292	-60.55%
**E153**	**G219**	**-61.80%**	L203	T293	-66.79%
**R165**	**P245**	**-53.87%**	I204	F291	-75.97%
**R165**	**L246**	**-72.36%**	N205	S260	-57.00%
**L166**	**L246**	**-87.99%**	V206	I290	51.08%
V186	M223	62.77%	D210	M259	-60.20%
G190	T306	50.04%	I214	I308	-53.44%
Q192	I225	-71.73%	M215	A310	79.44%
G193	M223	-73.77%	M215	T220	64.72%
G193	G224	-99.59%			

Supplemental Video Legends

Supplementary Video 1. Time evolution of the time-averaged structures at an interval of 10 ns of the GTP-bound MD simulations. The snapshots are superimposed on the initial structure.

Supplementary Video 2. Time evolution of the time-averaged structures at an interval of 10 ns of the GDP-bound MD simulations. The snapshots are superimposed on the initial structure.

Supplementary Video 3. Side views of the time evolution of the snapshots corresponds to **Video** 1. The main curvature places the C-terminus on the inside of the paper.

Supplementary Video 4. Side views of the time evolution of the snapshots corresponds to **Video** 2. The main curvature places the C-terminus on the inside of the paper.
